# Non-canonical Hh signalling through Smoothened controls cytotoxic T cell migration in the tumor microenvironment

**DOI:** 10.1126/sciimmunol.adr3127

**Published:** 2025-07-11

**Authors:** Chrysa Kapeni, Louise O’Brien, Dilyara Sabirova, Oliver Cast, Valentina Carbonaro, Stephen Clark-Leonard, Anne C. Machel, Flavio Beke, Sarah McDonald, Kate Fife, Maike de la Roche

**Affiliations:** 1 University of Cambridge, Cancer Research UK Cambridge Institute, Robinson Way, Cambridge CB2 0RE, UK; 2 Histopathology Department, Cambridge University Hospitals NHS Foundation Trust, Hills Road, Cambridge CB2 0QQ, UK; 3 Oncology Department, Cambridge University Hospitals NHS Foundation Trust, Hills Road, Cambridge CB2 0QQ, UK

## Abstract

The Hedgehog (Hh) signalling pathway is aberrantly regulated in cancer. Hh inhibitors are successful in treating basal cell carcinoma (BCC) and SHH-driven medulloblastoma but have largely failed in clinical trials of other solid cancers. We show that Hh inhibitor-treatment specifically diminishes CD8 T cell migration into the tumor microenvironment, both in murine cancer models and resected BCCs from patients treated with the Smoothened (SMO) inhibitor vismodegib. Using small molecule antagonists and genetic knockout models of key Hh signalling components, we demonstrate that the migration defect is mediated exclusively by the signal transducer Smo and not Hh ligands or Gli transcription factors. Smo acts non-canonically as a GPCR to regulate the migration of murine and human CD8 T cells via RhoA. Our data establishes the first link between Hh inhibition in vivo and the anti-tumor immune response and provides the basis for improved Hh targeting approaches for cancer patients.

## Introduction

The Hedgehog (Hh) pathway orchestrates cell fate choices throughout metazoan development and adult tissue homeostasis by regulating tissue patterning, cell proliferation and differentiation. In canonical signaling, extracellular Sonic, Desert or Indian Hh ligands (Shh, Dhh, Ihh) bind to the transmembrane receptor Patched (Ptch) on Hh-responsive cells. Upon binding, Ptch releases its inhibition of the transmembrane protein Smoothened (Smo). Smo translocates into the primary cilium, where it activates the glioma-associated oncogene (Gli) transcription factors, Gli1, Gli2 and Gli3. These move into the nucleus and initiate a Hh-specific transcriptional program^1^.

The pathway is also amplified in diverse human cancer types. Mutations in the pathway are oncogenic drivers in basal cell carcinoma (BCC)^2^, medulloblastoma^3, 4, 5, 6^ and a subset of rhabdomyosarcoma^7^. In addition, the pathway is aberrantly upregulated in many other human cancers, including pancreatic and colorectal adenocarcinoma as well as lung, breast, prostate, and hematological malignancies ^8^. As a result, Hedgehog inhibitors have emerged as promising anti-cancer therapeutics and clinically approved small molecule Hedgehog inhibitors have been the subject of multiple clinical trials (reviewed in^9^).

SMO inhibitors such as vismodegib and sonidegib (Erivedge and Odomzo, respectively) have shown excellent safety profiles in clinical trials^10, 11^ and have been approved by the FDA and EMA for the treatment of non-resectable advanced (both drugs) and metastatic (only vismodegib) BCCs. We collated the published results from trials that used the SMO antagonists vismodegib^12, 13^ and sonidegib^14^ to treat various cancer types (Table S1). Cancers with a Hh driver mutation, such as BCC and SHH medulloblastoma, benefited the most from Hh inhibition and showed remarkable response rates ranging between 8-71%. This was not the case in other cancers where, despite promising preclinical data, Hedgehog inhibitors have largely failed to meet primary endpoints in clinical trials of patients with colorectal^15^, pancreatic^16, 17, 18^ and prostatic adenocarcinomas^19^. Elucidating the mechanisms for the poor treatment outcomes is a high priority.

Although the Hedgehog pathway has been shown to be important for certain immune subsets during lymphopoiesis and autoimmune diseases^20, 21^, little is known about the effect of Hh inhibitors directly on CD8 cytotoxic T lymphocytes (CTLs). Previous work has shown that the pathway is required for CTL killing^22^. In T cells, the pathway is activated upon engagement of the T cell receptor (TCR) and controls actin clearance and centrosome polarization, a process essential for the formation of the immunological synapse and the targeted release of cytotoxic granules^22^. This work suggested that a reason for the lack of efficacy of Hh inhibitors in the clinic for many indications might be due to the inhibition of CTL killing, resulting in a diminished anti-tumor immune response. However, to date, no functional assessment of the anti-tumor T cell response during Hh inhibition *in vivo* in the tumor microenvironment has been performed.

In this study, we show that both pharmacological and genetic inhibition of Hh signaling *in vitro* and *in vivo* greatly impairs CTL migration into the tumor. Mechanistically, we show that the reduced CTL migration is not mediated by canonical Hh signaling and is therefore independent of the ligand Ihh and the transcription factor, Gli1, which is implicated in the formation of the immunological synapse. Instead, CTL migration in mouse and human is regulated non-canonically through the Hh signal transducer Smo and its G-protein coupled receptor (GPCR) function, which controls downstream levels of active RhoA. Finally, we show that migration of cytotoxic T cells into BCCs is diminished in patients treated with vismodegib.

## Results

### Tumor growth in a mouse model of colorectal cancer is exacerbated upon sonidegib treatment

**Figure 1:**
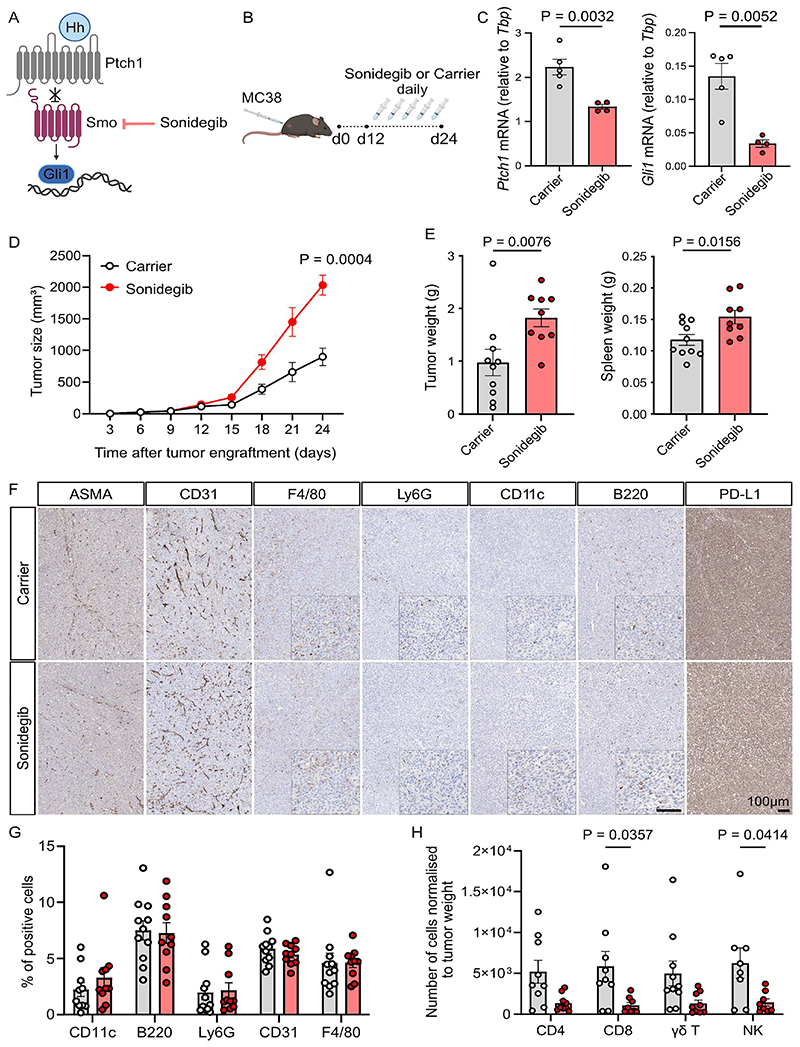
Treatment with clinically approved Hh inhibitor sonidegib exacerbates colorectal cancer growth. **(A)** Schematic of the canonical Hh signaling pathway. In the presence of Hh ligands, Ptch1 releases its inhibition of Smoothened (Smo), which leads to the activation of Gli transcription factors inducing a Hh-specific target gene program. Sonidegib is an FDA/EMA-approved Smo-specific antagonist. **(B)** Experimental Design. C57BL/6J wildtype mice were subcutaneously injected with 0.5 × 10^6^ MC38 colorectal tumor cells on d0. On d12, mice were stratified into two equal groups according to tumor size. Between d12 and d24, mice were treated daily with 20mg/kg sonidegib or carrier control by oral gavage. **(C)** mRNA levels of *Ptch1* and *Gli1* in the small intestine. One independent experiment, n=5 for carrier-treated mice, n=4 for sonidegib-treated mice, unpaired t-test, mean ± SEM. **(D)** Tumor growth was determined from caliper measurements. Two independent experiments shown, n=10 for carrier-treated mice, n=9 for sonidegib-treated mice, ordinary two-way ANOVA with Geisser-Greenhouse correction, mean ± SEM. **(E)** Tumor and spleen weight at endpoint (d24). Two independent experiments shown, n=10 for carrier-treated mice, n=9 for sonidegib-treated mice, unpaired Mann-Whitney test, mean ± SEM. **(F)** Representative images of MC38 tumors from mice treated with carrier (top row) or sonidegib (bottom row) and stained with antibodies against cell subsets as indicated. For F4/80, Ly6G, CD11c and B220 higher magnification inserts are shown in the bottom right corner. Scale bars equal 100 μm for both magnifications shown. **(G)** Quantification of cell subsets in the tumor microenvironment by IHC staining pictures shown in (**F**). Two independent experiments shown, n=11 for carrier-treated mice, n=10 for sonidegib-treated mice, two-way ANOVA, mean ± SEM. **(H)** Numbers of CD4, CD8, γδT cells and NK cells in tumor microenvironment assessed by flow cytometry and normalized to tumor weight. Two independent experiments shown, n=10 for carrier-treated mice, n=9 for sonidegib-treated mice, two-way ANOVA, mean ± SEM.

To investigate the lack of efficacy of Hh inhibitors in gastrointestinal cancers, we used a mouse model of colorectal adenocarcinoma. For this, we injected C57BL/6J mice subcutaneously with MC38^23^ tumor cells and let the tumor establish for twelve days. Mice were then stratified into two equal groups and treated daily by oral gavage with Smo inhibitor sonidegib or carrier control up to day 24 (Fig. 1A, B). Successful systemic inhibition of the Hh pathway *in vivo* was confirmed by downregulation of the key Hh target genes *Gli1* and *Ptch1*, in the small intestine and the skin (Fig. 1C, Fig. S1E). Strikingly, mice treated with sonidegib exhibited a doubling of tumor load as measured by size (Fig. 1D) and weight (Fig. 1E) and increased spleen weight.

### Sonidegib treatment does not lead to drastic changes in tumor composition

To assess whether sonidegib treatment led to global changes in the tumor microenvironment, we stained for fibroblasts (ASMA), endothelial cells (CD31), macrophages (F4/80), myeloid cells (Ly6G), dendritic cells (CD11c) and B cells (B220) in tumor tissue sections by IHC (Fig. 1F). Upon quantification, no significant differences were detected between sonidegib-treated versus carrier-treated tumor groups (Fig. 1G). We also assessed the tumor cell composition by analysing bulk RNASeq data using ConsensusTME^24^ (Fig. S1A). The analysis confirmed no significant changes in the tumor stroma, blood vessels or in the myeloid cell, B cell, and dendritic cell compartments. In addition, sonidegib treatment did not affect the composition of peripheral blood of treated mice (Fig. S1F).

### Cytotoxic T cell infiltration into the tumor is diminished upon sonidegib treatment

Despite no observational change in the overall tumor composition, a significant reduction in the infiltration of predominantly cytotoxic lymphocytes into the tumor mass was detected by flow cytometry (Fig. 1H, Fig. S1D) and bulk RNASeq (Consensus TME) (Fig. S1A). Cytotoxic CD8 T cells and NK cells in the TME of sonidegib-treated mice were 81% and 77% reduced, respectively. The levels of CD4 T cells and gamma delta T cells were also moderately reduced.

**Figure 2:**
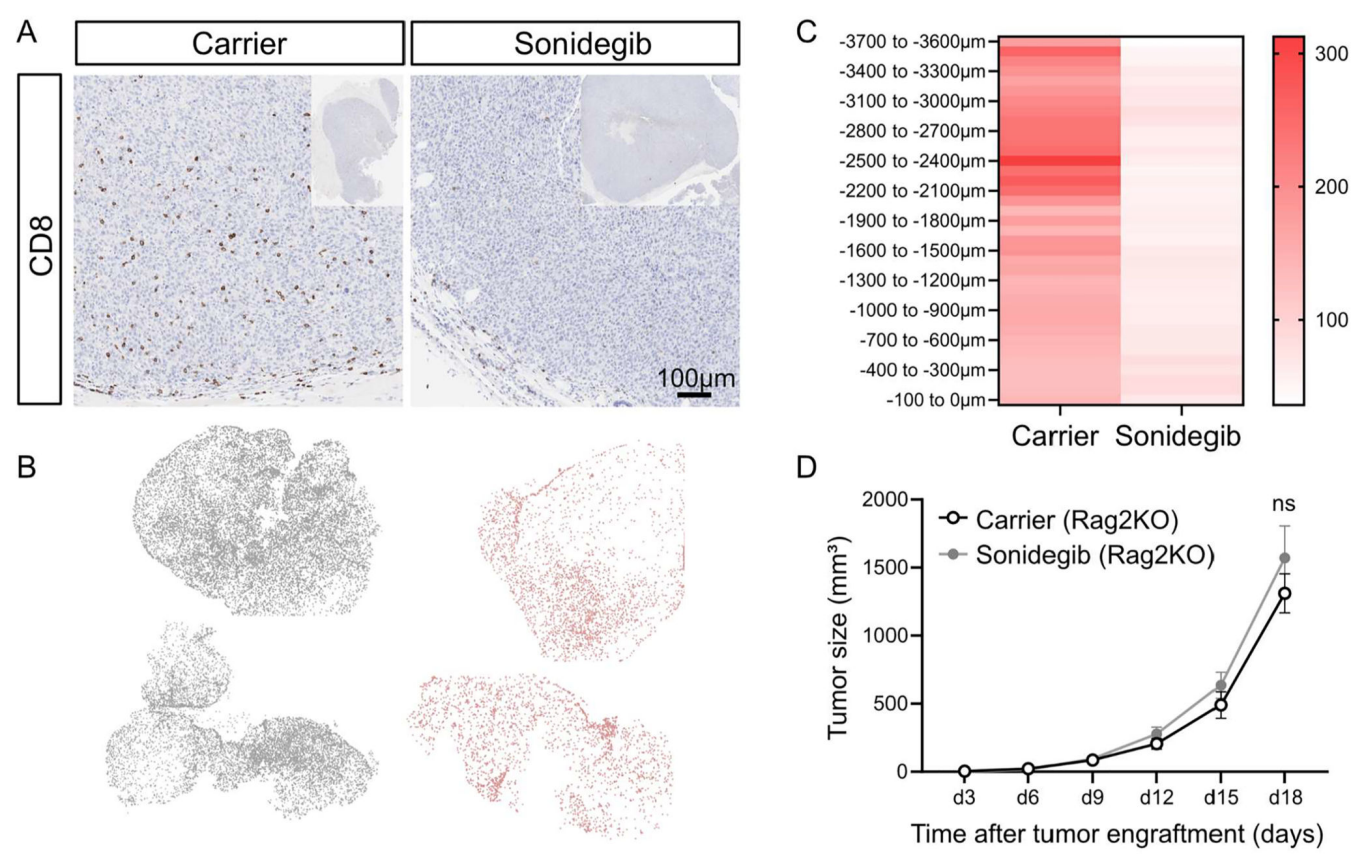
Sonidegib treatment inhibits CD8 migration into the tumor. **(A)** Representative paraffin sections from MC38 tumor-bearing mice treated with either carrier control or sonidegib and stained with an anti-mouse CD8a antibody. The whole tumor is shown in top right insert panels. **(B)** HALO analysis of CD8 cell infiltration for two representative tumors of carrier-treated (grey) and sonidegib-treated (red) mice, respectively. Each dot represents a CD8 cell. **(C)** Mean of number of CD3+CD8+ cells/ mm^2^ in 100μm-wide zones from the tumor surface (-100 to 0 μm) to the tumor centre (-3700 to -3600 μm) as quantified by HALO software (analysis workflow shown in Fig. S2A). Two independent experiments shown, n=10 for carrier-treated and n=8 for sonidegib-treated mice. **(D)** Tumor size determined from caliper measurements. *Rag2*KO mice were subcutaneously injected with 0.5 × 10^6^ MC38 cells on d0. On d9, mice were stratified into two equal groups according to tumor size. Between d9 and d18, mice were treated daily with 20mg/kg sonidegib or carrier control by oral gavage. Two independent experiments shown, n=7 for carrier-treated tumors, n=7 for sonidegib-treated tumors, two-way ANOVA, mean ± SEM.

To investigate this further, we performed IHC staining for CD8 cells on tumor sections (Fig. 2A, B). To assess the extent of CD8 T cell infiltration into the tumor mass, we used the imaging software HALO to segment the tumor in 100 micron-wide concentric zones (Fig. S2A). Our analysis showed that not only were there more CD8 T cells (CD3+, CD8+) in carrier-treated tumors, but the CD8 T cells were also able to travel further towards the tumor center compared to sonidegib-treated tumors (Fig. 2C). This was also true for total CD3 T cells (Fig. S2B, C).

To exclude the possibility that the cytotoxic cells in tumor-bearing mice were specifically retained in secondary lymphoid tissues such as spleen and tumor-draining lymph nodes, we assessed cytotoxic cell numbers in these organs by flow cytometry. No significant change in the numbers of CD8, CD4, gamma delta T cells or NK cells was observed upon Hh inhibitor treatment in the spleen (Fig. S1B). A moderate reduction in both CD4 and CD8 T cell numbers was noted in the tumor-draining lymph node, 31% and 32%, respectively (Fig. S1C). These data suggests that the reduction of cytotoxic lymphocytes in the tumors of sonidegib-treated mice was not due to a retention of the cells in secondary lymphoid organs.

To further investigate whether the loss of tumor control (Fig. 1D) was due to an effect of Hh inhibitors on cytotoxic T cells or NK cells, we systemically inhibited Smo in RAG2KO mice which lack B and T lymphocytes but retain NK cells. Tumors in RAG2KO mice grow faster due to the lack of T cells, so we adopted an earlier endpoint on day 18 (Fig. 2D). We observed no difference in tumor growth upon Smo inhibition indicating the exacerbated tumor growth observed in C57BL/6J mice is driven by a repressive effect of sonidegib on adaptive T lymphocytes rather than NK cells.

In summary, our data show that treating immunocompetent tumor-bearing mice with Smo inhibitors results in fewer cytotoxic T cells in the tumor with compromised ability of the cells to infiltrate deeply into the tumor mass likely leading to impaired tumor rejection.

### Genetic ablation of *Smo* in CD8 T cells results in reduced CD8 infiltration in the tumor and increased tumor load

To confirm that the observed lack of infiltration and tumor control in the Hh-inhibited animals was due to a direct and specific effect of the inhibitors on CD8 T cells and not due to indirect effects via other cell types or off-target effects, we generated a conditional knockout mouse line. In this line, *Smo* is floxed and can be inducibly deleted selectively in cytotoxic lymphocytes via an *ERT2Cre* recombinase under control of the granzyme B promoter, circumventing any effects of *Smo* loss on T cell development. A tdTomato fluorescence reporter was included to detect Cre-mediated recombination.

**Figure 3:**
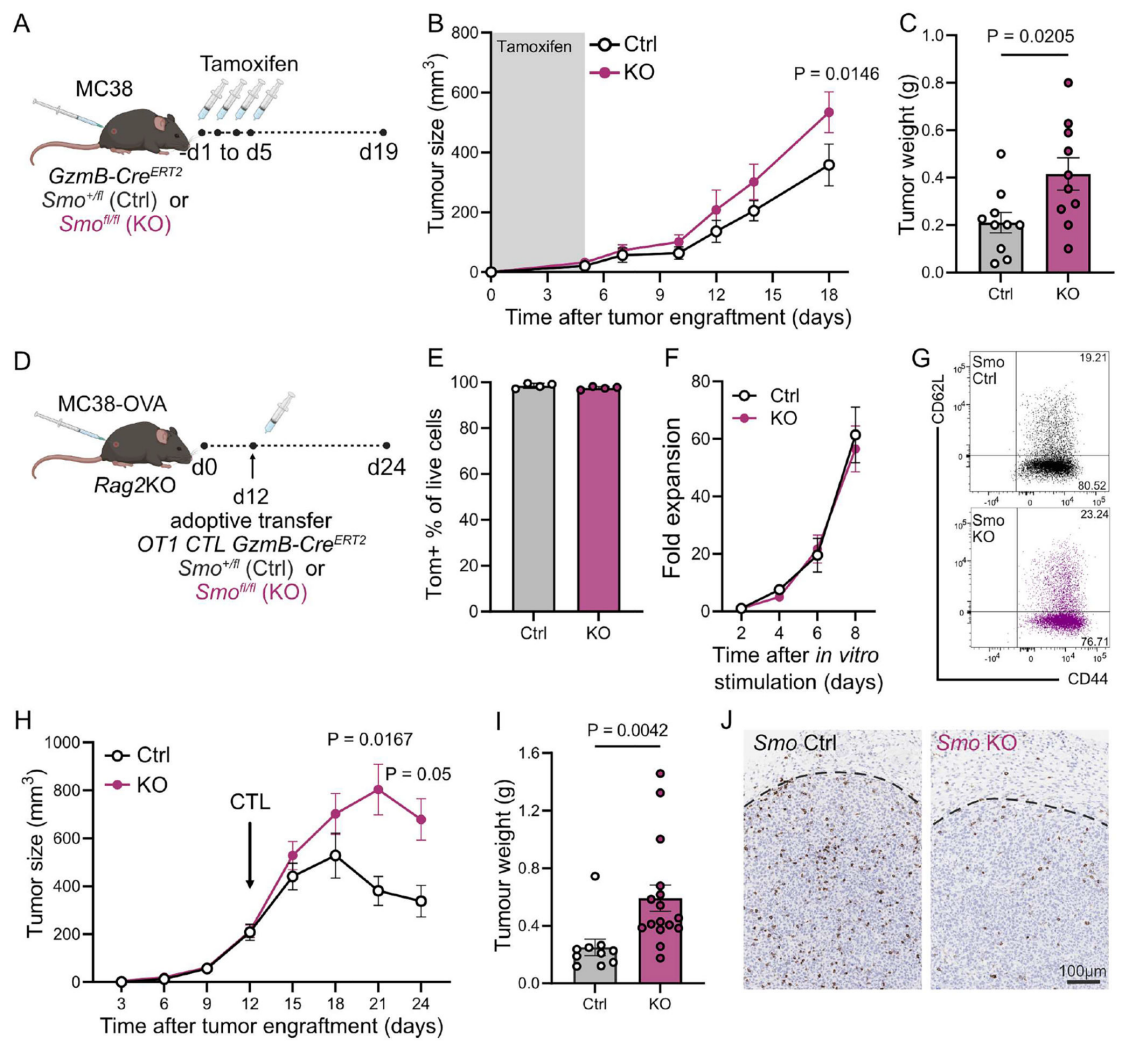
Genetic loss of *Smo* in cytotoxic T cells increases tumor growth. **(A)** Experimental Design. *GzmB-ERT2Cre/ROSAtdTom/Smo*^*fl/fl*^ (KO) or *Smo*^*fl/+*^ (Ctrl) animals were subcutaneously injected with 0.5 × 10^6^ MC38 cells on d0. Mice were treated with tamoxifen via intraperitoneal injections (75mg/kg) on -d1, d1, d3 and d5. **(B)** Tumor dimensions determined from caliper measurements. Four independent experiments, n=11 for *Smo*^*fl/+*^ (Ctrl), n=10 for *Smo*^*fl/fl*^ (KO), 2-way ANOVA, mean ± SEM. **(C)** Tumor weight at endpoint (d19). Four independent experiments shown, n=10 for *Smo*^*fl/+*^ (Ctrl), n=10 for *Smo*^*fl/fl*^ (KO), unpaired t-test, mean ± SEM. **(D)** Experimental Design. *Rag2*KO animals were subcutaneously injected with 0.5 × 10^6^ MC38-OVA cells on d0. In parallel, T cells from *GzmB-ERT2Cre/ROSAtdTom*-*OTI* mice either control or *Smo* knockout were activated with OVA peptide for 48hrs and expanded in the presence of OHT for 6-8 days. On d12, tumor-bearing mice were stratified into two equal groups according to tumor size and 1 × 10^6^ of either control (Ctrl) or Smo knockout (KO) cytotoxic T cells (CTLs) were adoptively transferred by tail vein injection. **(E)** Percentage of Tom+ cells on d7 of *ex vivo* T cell expansion, n=4 for *Smo*^*fl/+*^ (Ctrl), n=4 for *Smo*^*fl/fl*^ (KO), mean ± SD. **(F)** Fold expansion of CD8 cells *in vitro* following 48h stimulation with OVA peptide in the presence of OHT. Four independent experiments shown, n=7 for *Smo*^*fl/+*^ (Ctrl), n=8 for *Smo*^*fl/fl*^ (KO), mean ± SEM. **(G)** Representative flow cytometry plots of CD62L/CD44 expression on CTLs from d7 of expansion that were subsequently used for the adoptive transfers (**D, H-J** of this figure). **(H)** Tumor dimensions were determined from caliper measurements. Four independent experiments shown, n=10 for *Smo*^*fl/+*^ (Ctrl), n=16 for *Smo*^*fl/fl*^ (KO), ordinary two-way ANOVA with Geisser-Greenhouse correction and Sidak’s multiple comparison tests, mean ± SEM. **(I)** Tumor weight at endpoint (d24). Two independent experiments shown, n=10 for *Smo*^*fl/+*^ (Ctrl), n=16 for *Smo*^*fl/fl*^ (KO), unpaired Mann-Whitney test, mean ± SEM. **(J)** Representative paraffin sections from MC38-OVA tumors shown in **(H)** and stained with anti-mouse CD8α antibodies. Dotted line indicates tumor margins.

MC38 tumors were subcutaneously implanted in *Smo* HET (Ctrl, *GzmB-ERT2Cre/ROSAtdTom/Smo*^*+/fl*^) and KO (*GzmB-ERT2Cre/ROSAtdTom/Smo*^*fl/fl*^) mice and excision of *Smo* in cytotoxic lymphocytes was induced by administration of tamoxifen. Tamoxifen was given one day before tumor implantation and then again on d1, d3 and d5 post implantation (Fig. 3A). *Smo* KO mice exhibited accelerated tumor growth compared to HET animals, resulting in a 2-fold increase in tumor weight at experimental endpoint (Fig. 3B, C). At endpoint, the tumors in the *Smo* KO mice also harboured reduced numbers of CD8 and NK cells, albeit no statistically significant differences were observed (Fig. S3A). These experiments indicate that genetic ablation of *Smo* specifically in cytotoxic lymphocytes also results in higher tumor burden, similarly to sonidegib-treated animals.

The above tumor model had the following limitations: firstly, using this schedule of tamoxifen treatment, only an average of 40% of cytotoxic lymphocytes was excised (as determined by expression of the tdTomato fluorescent Cre reporter) (Fig. S3A); secondly, the model did not allow us to track the tumor antigen-specific T cell response; and thirdly, the *GzmB-ERT2Cre* also induces recombination in GzmB+ cytotoxic CD4+ T cells, gamma delta T cells and NK cells^25^. Thus, we modified our experimental design to exclusively assess the functional CD8 T cell anti-tumor response. For this purpose, we crossed our mouse line to the OTI transgenic strain to generate CD8 *Smo* KO tumor-specific CTLs *in vitro* and adoptively transferred the cells into MC38-OVA tumor-bearing, immunodeficient *Rag2KO* recipient mice. With this setup we can interrogate the role of Smo in tumor-specific T cells and without potential effects of *Smo* deletion on CTL priming, expansion, and differentiation (Fig. 3D).

CD8 T cells from Ctrl and *Smo* KO mice were stimulated with OVA and treated with OH-tamoxifen *ex vivo* before being transferred into tumor-bearing mice. *In vitro*, Smo deletion was confirmed by qRT-PCR (Fig. S3B) and although more than 97% of the cells were excised by d7 of culture (Fig. 3E), CD8 T cell expansion or effector phenotype were not affected (Fig. 3F, G). *In vivo*, genetic ablation of *Smo* in tumor-specific CD8 T cells lead to a doubling of the tumor burden (Fig. 3H, I) and diminished T cell infiltration into the tumor mass (Fig. 3J), recapitulating the effect of sonidegib treatment.

### Genetic ablation of *Ihh* and *Gli1* in CD8 T cells does not result in reduced tumor infiltration or increased tumor load

Smo is the main signal transducer of the Hh pathway and the molecular target of almost all clinically used inhibitors, including vismodegib and sonidegib. To investigate mechanistically whether the migration phenotype we observed was perhaps regulated by Hh ligands upstream or the Gli transcription factors downstream of Smo (Fig. 1A), we assessed CD8 T cells deficient in either *Indian Hh* (*Ihh*) or *Gli1*. Previous work from our laboratory has shown that CD8 CTLs only express Ihh ligand (and not Dhh or Shh)^22^ and effector CD8 T cells are unable to respond to exogenous Hh ligands^22^. Furthermore, Gli1 is the main transcription factor expressed in CD8 CTLs^22^.

OTI-CTLs deficient in *Ihh* or *Gli1* were generated *ex vivo* from our respective mouse models and adoptively transferred into MC38-OVA tumor-bearing *Rag2KO* recipient mice. Loss of *Ihh* or *Gli1* in CD8 T cells did not alter proliferation or effector phenotype *in vitro* (Fig. S3G, H, M, N).

*In vivo*, OTI-CTLs deficient in *Ihh* or *Gli1 were* equally effective as their wildtype counterparts at controlling tumor growth (Fig. S3G, M). While tumor weights were identical in the mice treated with *Ihh* Ctrl versus KO OTI-CTLs, we observed a reduced, albeit non-significant, ability of *Gli1*KO OTI-CTLs to control MC38-OVA tumors *in vivo* (Fig. S3M, N). Furthermore, the levels of tumor infiltration by CD8 T cells were unaffected by the loss of either *Ihh* or *Gli1* in OTI-CTLs (Fig. S3I, O).

These data show that, unlike genetic ablation of *Smo*, loss of *Ihh* or *Gli1* in cytotoxic CD8 T cells, does not affect tumor growth or restrict CD8 migration into the tumor microenvironment in the models tested.

### Pharmacological inhibition or genetic ablation of *Smo*, but not *Ihh* or *Gli1*, inhibits CTL migration towards tumor cells

**Figure 4:**
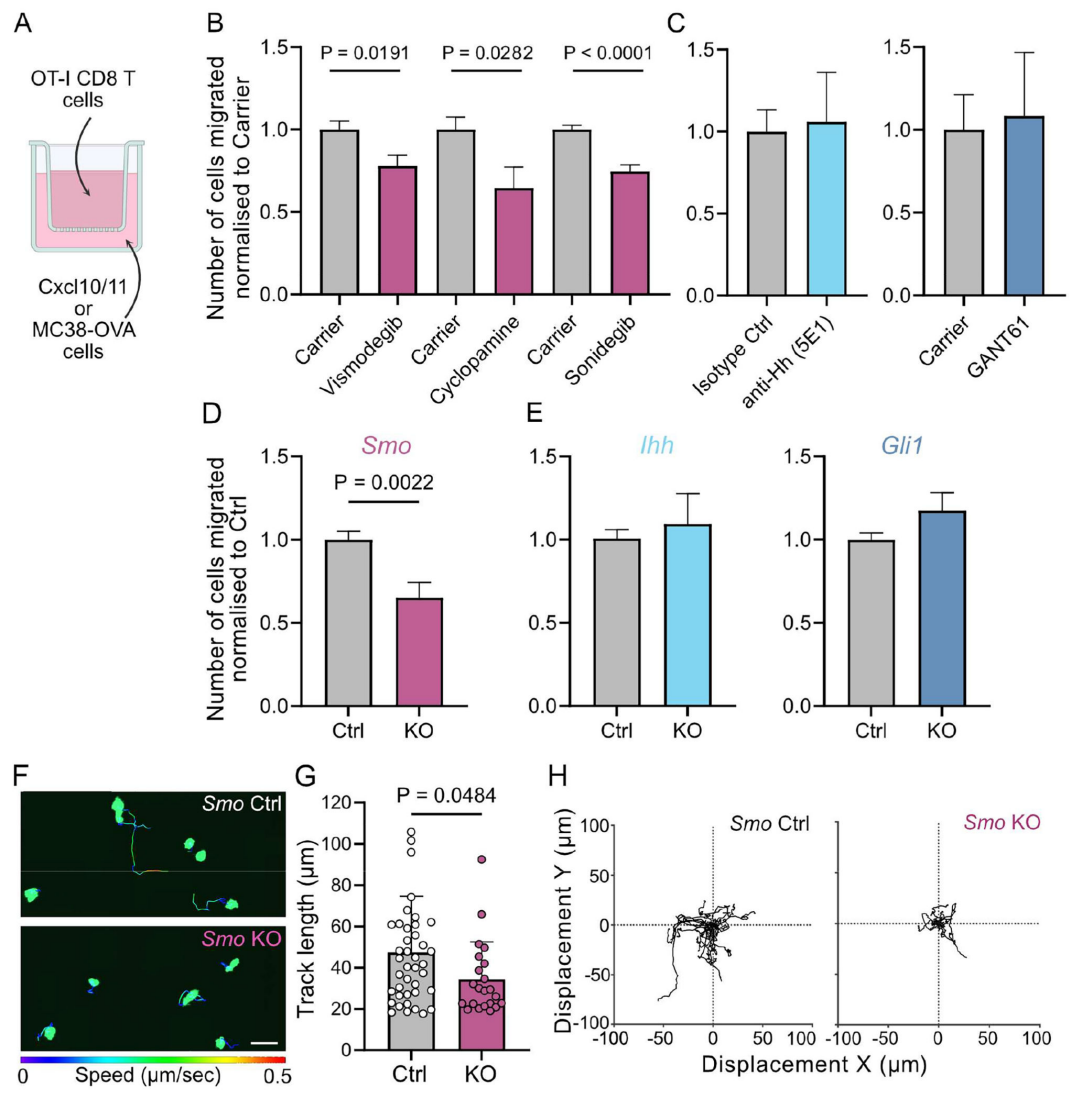
Loss of *Smo*, but not *Ihh* or *Gli1*, inhibits CD8 migration *in vitro*. **(A)** Schematic of transwell experiment to assess directed migration. Cxcl10 and Cxcl11-supplemented T cell media **(B, C)** or MC38-OVA cells **(D, E)** were supplied in the bottom well. A 5μm transwell insert was placed on top and CTLs on d6-8 of culture were dispensed in the insert. After 6hrs, cells from the bottom well were collected for flowcytometric analysis. **(B)** T cell migration in the presence of Smo inhibitors. Vismodegib, n=6, two independent experiments shown; Cyclopamine, n=6, two independent experiments shown, and Sonidegib, n=8, two independent experiments shown. For each drug, paired t-tests were performed, and mean ± SEM is shown. **(C)** T cell migration in the presence of Hh ligand blocking antibody 5E1 and Gli inhibitor GANT61. 5E1: n=4, two independent experiments shown, GANT61: n=3, two independent experiments shown. For each drug, paired t-tests were performed, and mean ± SEM is shown. **(D)** CTLs were generated from conditional *Smo*^*fl/+*^*or Smo*^*+/+*^ (Ctrl) as well as *Smo*^*fl/fl*^ (KO) mice and migratory capacity was assessed. Migration is normalised to Ctrl. n=4 for Ctrl, n=4 for KO. All samples were run in technical triplicates across three independent experiments (all shown together). Unpaired t-test, mean ± SEM. **(E)** CTLs were generated from conditional *Ihh*^*fl/+*^ (Ctrl) or *Ihh*^*fl/fl*^ (KO) mice as well as *Gli1*^*+/+*^ (Ctrl) or *Gli1*^*eGFP/eGFP*^ (KO) mice and migratory capacity was assessed. Migration is normalized to Ctrl. n=4 for *Ihh* Ctrl, n=5 for *Ihh* KO, n=5 for *Gli1* Ctrl and n=5 for *Gli1* KO. All samples were run in technical triplicates across three to four independent experiments (all shown together). Unpaired t-test, mean ± SEM. **(F-H)** CTLs were generated from conditional *Smo*^*fl/+*^*or Smo*^*+/+*^ (Ctrl) as well as *Smo*^*fl/fl*^ (KO) mice and migratory capacity of cells was assessed by live imaging on Icam-coated glass slides for 20min. **(F)** Representative snapshots of live imaging with individual cell tracks shown. Track gradient denotes speed (blue to red, 0 to 0.5 μm/sec) and scale bar equals 20 microns. **(G)** Mean track length of CFSE-labelled *Smo* Ctrl and KO CTLs is shown. Each dot represents an individual cell. n=4 *Smo* Ctrl and n=4 *Smo* KO, two independent experiments (one representative experiment shown). Unpaired t-test, mean ± SD. **(H)** XY displacements graphs for representative samples shown in **(F)**.

To investigate whether the diminished MC38 tumor infiltration by CD8 T cells seen upon sonidegib treatment and genetic ablation of *Smo* in the T cells was indeed caused by reduced migratory capacity, we utilized a transwell migration assay. Here, MC38-OVA cells were plated in the bottom well and OTI-CTLs were added to the insert (Fig. 4A).

Addition of Smo inhibitors, including the clinically approved vismodegib and sonidegib as well as the *bona fide* Smo inhibitor cyclopamine reduced CTL migration by 20-40% (Fig. 4B). By contrast, treating the CTLs with a 5E1 antibody that blocks Hh ligands (Ihh, Shh, Dhh) from binding to Ptch1, or the Gli1 antagonist GANT61, had no effect on CTL migration (Fig. 4C). All inhibitors tested did not affect T cell viability (Fig. S4K).

Taken together the data is consistent with our *in vivo* findings using *Ihh* and *Gli1* KO T cells (Fig. S3E-P), suggesting the migration effect is mediated by Smo.

To confirm that the reduced migration observed in the presence of Smo inhibitors was indeed Smo-specific and not caused by toxicities, we performed transwell assays using *Smo* KO OTI-CTLs. We observed a 35% reduction of the ability of *Smo* KO CTLs to reach the tumor cells compared to *Smo* Ctrl CTLs (Fig. 4D). Genetic deletion of either *Ihh* or *Gli1* again did not affect the CTL migration in the *in vitro* system (Fig. 4E).

To further investigate Smo-dependent CTL migratory capacity in real-time, we developed a live cell imaging system. Here, we use a spinning disk confocal microscope to track CTL migration on ICAM-coated glass over 20 minutes. *Smo* KO CTLs displayed reduced speed (Fig. 4F) with overall 27% reduced track length (Fig. 4G) resulting in lower displacement from their starting point compared to *Smo* WT cells (Fig. 4H).

Our *in vitro* migration assays reveal that Smo, but not canonical Hh signaling through Ihh or Gli1, is critical for the migration of CTLs towards tumor cells.

### Smo regulates CTL migration via its GPCR function

We have shown that Smo is critical for CTL migration *in vitro* and *in vivo* and acts independently of both the Hh ligand Ihh and the transcription factor Gli1. T cell migration into the tumor microenvironment is guided by chemokine gradients. A small set of chemokines are secreted by tumor cells and are recognised by chemokine receptors expressed on T cells^26^.

We were interested to investigate whether Smo might broadly regulate the expression of chemoattractants and receptors in T cells. We first profiled cytokine and chemokine expression in *Smo* WT and KO CTLs using a XL-cytokine and chemokine array (Fig. S4A, B). Out of 111 targets probed, only few were expressed in CTLs (Fig. S4A), and quantification of the cytokines and chemokines expressed did not show significant differences between *Smo* wildtype and *Smo* KO cells (Fig. S4B). In addition, mRNA expression of the top hits from the array was also not significantly altered upon loss of *Smo* (Fig. S4C).

We next determined the main chemokines expressed by the tumor cells used in our *in vitro* migration assays and the tumor-bearing mice models. MC38-OVA cells express high levels of Cxcl10 and lower levels of Cxcl11 and Cxcl9. Ccr1, 3, 5, Cxcl12 and Cxcr4 were barely detected (Fig. S4D). All three expressed cytokines, Cxcl9, 10, and 11 bind to the Cxcr3 receptor. Thus, we profiled Cxcr3 expression in Ctrl or *Smo* KO CTLs by qRT-PCR (Fig. S4E), flow cytometry and immunoblotting (Fig. S4F, G). The loss of *Smo* did not lead to any significant alterations in Cxcr3 expression. To investigate whether the Cxcr3-Cxcl9/10/11 signaling axis is active in the transwell migration assay, we treated CTLs with either the Cxcr3 antagonist SCH546738 or a blocking antibody against Cxcr3 (α-Cxcr3). Both treatments resulted in a reduction of CTL migration by 31% and 20%, respectively, when WT cells were used in the migration assay (Fig. S4H). When treating *Smo* KO cells with SCH546738 or α-Cxcr3, we did not observe a synergistic effect between Smo and Cxcr3 (Fig. S4I, J). Taken together, these data suggest that genetic ablation of *Smo* in CTLs has no effect on the CTL chemokine profile, and although CTLs still utilize the Cxcr3-Cxcl9/10/11 axis for migration, this dependency is not differentially regulated in *Smo* KO CTLs.

**Figure 5:**
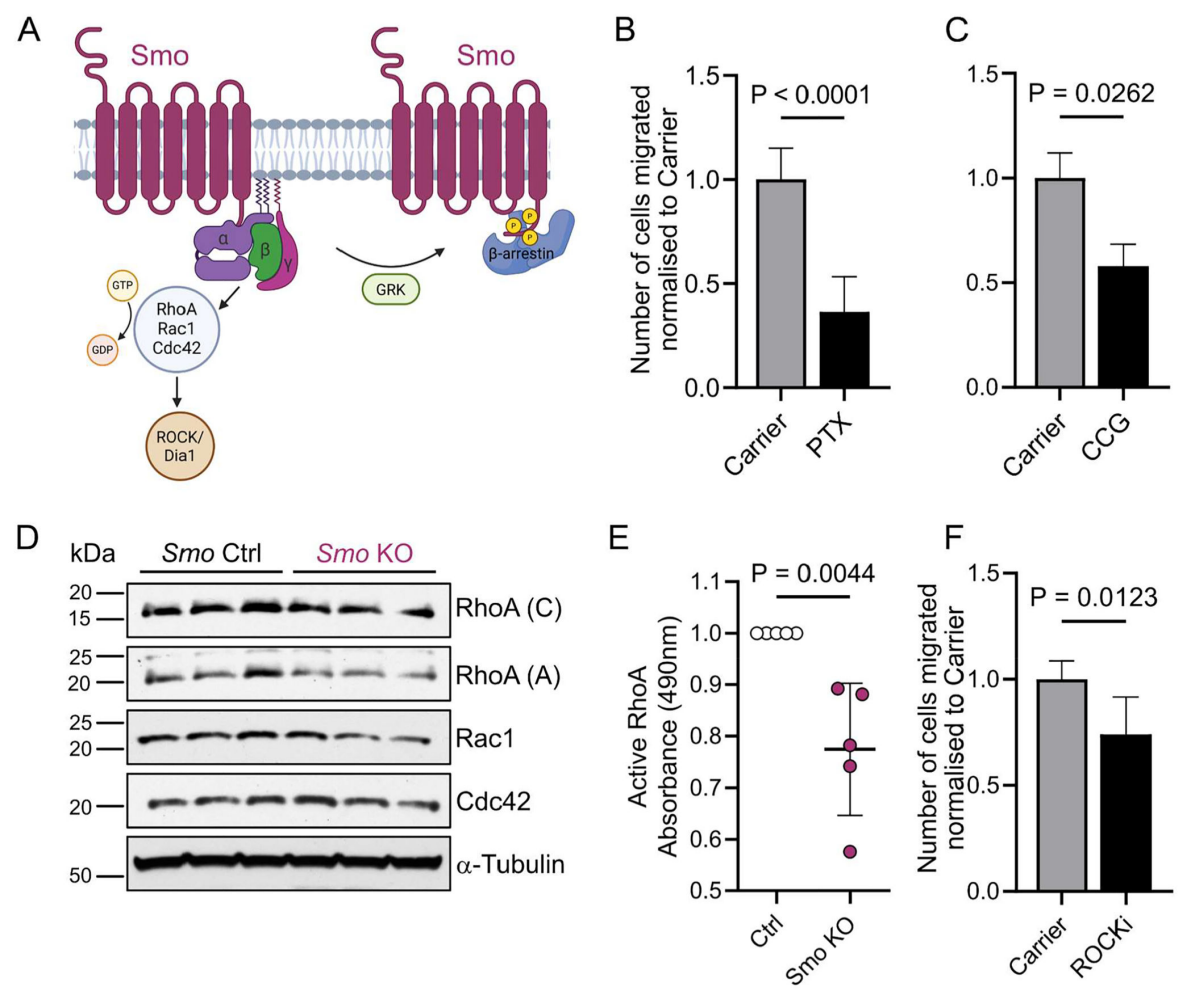
Genetic loss of *Smo* affects cytotoxic CD8 migration via GPCR coupling. **(A)** Smoothened (Smo) is a class F 7-TM G-protein coupled receptor. Smo has been shown to utilize Gαi heterotrimeric proteins and regulate the activation of Rho GTPase subfamily members, RhoA, Rac1 and Cdc42. After GPCR activation, GRKs regulate the phosphorylation of the Smo C-terminus and recruitment of arrestins^27^. Created in BioRender. Kapeni, C. (2025) https://BioRender.com/2a5r7oe. **(B, C)** Transwell migration assays with Cxcl10 and Cxcl11-supplemented T cell media at the bottom well. CTLs on d6-8 of culture in drug-containing media were dispensed in the insert. After 6hrs, cells from the bottom well were collected for flow cytometric analysis. *Bortedella pertussis* toxin (PTX) 100ng/ml, five independent experiments shown, n=13. CCG215022 0.32mM, two independent experiments shown, n=6. All samples were run in technical quadruplicates. For each drug paired t-tests were performed and mean ± SD is shown. **(D, E)** CTLs were generated from conditional *Smo*^*fl/+*^*or Smo*^*+/+*^ (Ctrl) as well as *Smo*^*fl/fl*^ (KO) mice. **(D)** Representative protein immunoblot analysis of *Smo*^*fl/+*^ (Ctrl) and *Smo*^*fl/fl*^ (KO) CTL lysates. Two separate antibodies were tested for RhoA, one from Cytoskeleton (**C**) and one from Abcam **(A)**. Three independent experiments (one representative experiment shown), n=6 for *Smo* Ctrl and n=6 for *Smo* KO, quantification in Fig. S5C. **(E)** Levels of active, GTP-loaded RhoA measured by absorbance set at 490nm with an ELISA-based assay. Three independent experiments shown, n=5 for Smo Ctrl and n=5 for Smo KO, unpaired t-test, mean ± SD. **(F)** Transwell migration assays as in **(B, C)**. ROCK inhibitor 1 μM, two independent experiments shown, n=5, paired t-test mean ± SD.

Apart from being the key signal transducer for canonical Hh signaling, Smo has been shown to function as a class F G-protein coupled receptor (GPCR) (reviewed in^27^) (Fig. 5A). Importantly, previous work in fibroblasts has shown that Smo can regulate migration via its GPCR function^28^. To determine whether a similar effect may be at play in CTLs, we blocked coupling of Smo to G_i_ subunits by treating the cells with pertussis toxin (PTX). PTX blocks G protein-GPCR interactions by catalyzing the ADP-ribosylation of α_i_ subunits^29^. Notably, PTX led to a 74% reduction in CTL migratory capacity (Fig. 5B, Fig. S5A).

Other studies have suggested that after Smo activation, the Smo c-tail can become phosphorylated, leading to an altered conformation permissive to β-arrestin recruitment. Recruitment of β-arrestin to the active conformation can then drive high-level GPCR signal propagation and eventual Smo desensitization^27^. To determine if c-tail phosphorylation and arrestin recruitment could play a role in CTL migration, we used a G protein-coupled receptor kinase (GRK) inhibitor, CCG215022, to block kinase activity, inhibiting GPCR phosphorylation. Treating CTLs with the GRK inhibitor resulted in a 43% decrease in CTL migratory capacity (Fig. 5C, Fig. S5A).

To understand the mediators of T cell migration downstream of Smo GPCR activity, we turned to RhoA, a small GTPase which has been shown to regulate fibroblast migration^28^. First, we confirmed expression of RhoA and other small GTPases including Rac1 and Cdc42 at steady state in CTLs by bulk RNAseq (Fig. S5B). We then compared protein levels of total RhoA, Cdc42, and Rac1 in *Smo* Ctrl and KO CTLs which were not significantly altered (Fig. 5D, Fig. S5C). However, expression of RhoA and Rac1 were marginally reduced in the KO.

Next, we assessed the effect of *Smo* loss on active RhoA. Intriguingly, levels of GTP-bound, active RhoA in *Smo* KO CTLs were reduced by 22% (Fig. 5E). In addition, CTLs treated with a Rho kinase inhibitor, displayed 26% reduced migration compared to vehicle-treated controls (Fig. 5F, Fig. S5A).

Taken together, our data suggests that CTLs rely on GPCR coupling, RhoA and c-tail phosphorylation to migrate. CTLs with genetic ablation of *Smo* have lower levels of active RhoA, resulting in diminished migratory capacity.

### Vismodegib treatment reduces T cell infiltration in basal cell carcinoma

**Figure 6:**
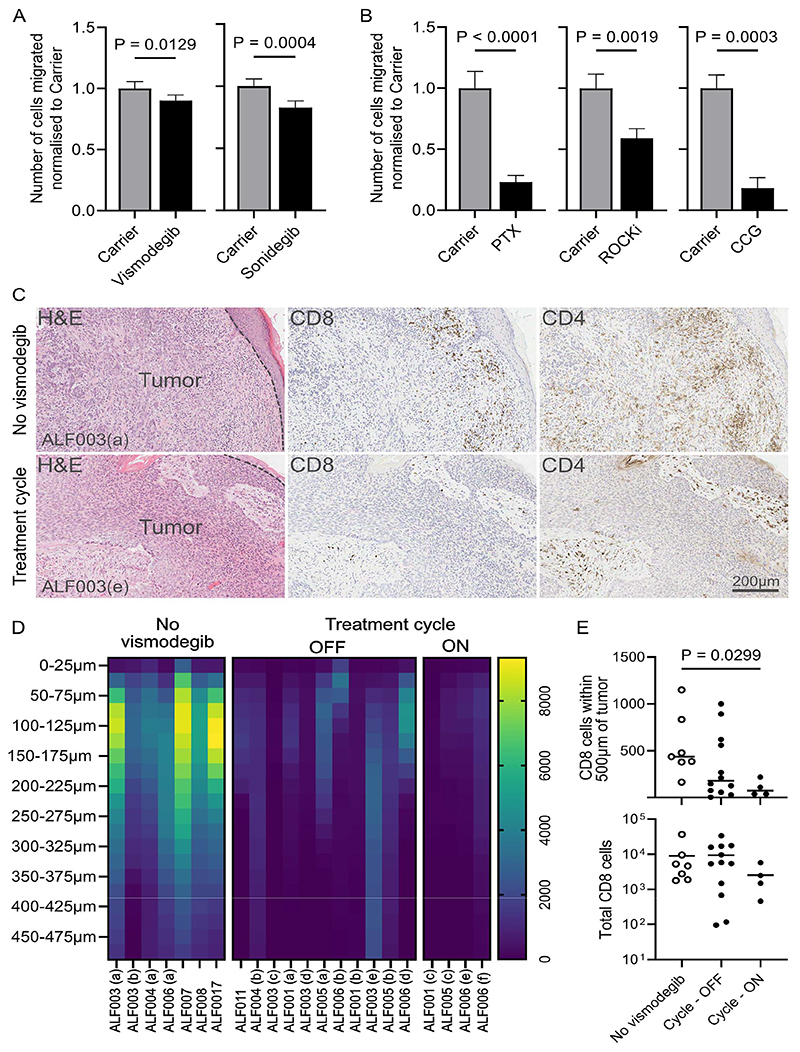
Inhibition of SMO diminishes human CD8 T cell migration *in vitro* and in the clinic. **(A)** Transwell assays with CXCL10 and CXCL11-supplemented T cell media at the bottom well. Inserts coated with matrigel were placed on top of the well and d10-12 CTLs were dispensed in the insert in drug-containing media. Vismodegib 5 μM, two independent experiments shown, n=10 healthy donors. Sonidegib 5-10 μM, two independent experiments shown, n=10 healthy donors. For each drug paired t-tests were performed and mean ± SD is shown. **(B)** Transwell assays as in **(A)**. *Bortedella pertussis* toxin (PTX) 200ng/ml, two independent experiments shown, n=9 healthy donors. ROCK inhibitor 10 μM, two independent experiments shown, n=6 healthy donors, GRK inhibitor CCG215022 0.032-0.32mM, three independent experiments shown, n=11 healthy donors. For each drug, paired t-tests were performed and mean ± SEM is shown. **(C)** Representative sections of BCCs resected from the same patient either before vismodegib treatment, sample ALF003 (a) (*top row*) or during vismodegib treatment cycle, sample ALF003 (e) (*bottom row*). *Left panel:* H&E staining, dotted lines demarcate tumor margins; *middle panel*: CD8 staining, *right panel*: CD4 staining. Scale bar equals 200 μm. **(D)** Proximity analysis of each BCC cell to its closest neighboring CD8 T cell (analysis workflow shown in Supp. Fig. 6C-E). Heatmap indicates the number of BCC tumor cells within the indicated distance brackets. n=7 BCCs before vismodegib treatment, n=11 BCCs during treatment cycle (“off”) and n=4 BCCs during treatment cycle (“on”), originating from nine patients in total. Samples are presented in chronological order, BCCs during treatment cycle (“off”) are ordered according to length of time since last vismodegib dose, from longest (*left*) to shortest (*right*). Detailed dates of sample collection, excision site, duration of treatment regime and type of tumor is shown in Table S2. **(E)** Numbers of CD8 T cells found within 500 μm of the tumor margins (*top*) and total numbers of CD8 T cells across the entire tumor section (*bottom*) in the same excisions shown in **(D)**. Unpaired t-test was performed, and mean is shown.

To determine whether human CTLs also rely on SMO to migrate, we performed transwell assays with SMO inhibitors vismodegib and sonidegib. The migratory capacity of human CD8 T cells isolated from healthy donors was reduced in the presence of vismodegib by 14% and sonidegib by 21% (Fig. 6A). Both inhibitors did not affect T cell viability at the concentrations used (Fig. S6A).

We then treated human CD8 T cells with PTX, Rho kinase and GRK inhibitors, to establish whether, like in murine CD8 T cells, the GPCR machinery was driving human CTL migration. Human T cells were even more sensitive to all three drugs compared to murine T cells, leading to a reduction in migratory capacity by 77% (PTX), 41% (ROCKi) and 82% (GRKi), respectively (Fig. 6B). The viability of primary human T cells was unaffected by inhibitor treatment (Fig. S6B).

To assess if our findings apply to patients treated with SMO inhibitors in the clinic, we studied cancer patients with multiple basal cell carcinomas (BCCs). A small subset of patients with locally advanced or metastatic BCCs who cannot be treated with surgery or radiation are systemically treated with the SMO inhibitor vismodegib in the UK. We initiated a clinical research study at Addenbrooke’s Hospital (Altered Lymphocyte Function in health and disease, ALF study) and recruited BCC patients treated with vismodegib in an *“on”*/*“off”* treatment cycle pattern, typically lasting three months (Table S2).

We sought to determine whether the number of CD8 T cells in BCCs was affected by treatment with vismodegib. We collected 23 rare tumor biopsies in total, some originating from the same patients several years apart, taken before vismodegib treatment started and after patients were placed on a vismodegib treatment cycle either when *“off”* or *“on”* treatment (Table S2). As observed in our murine cancer models, infiltration of CD8 T cells into the TME was reduced upon treatment with SMO inhibitor shown by immunohistochemistry (Fig. 6C).

CD8 T cells need to be in close/direct contact to tumor target cells to efficiently carry out their function^30^. Thus, we next calculated the distance between each BCC cell and its nearest CD8 T cell (workflow shown in Fig. S6C-E). When assessing the distance between CD8 T cells and tumor cells, in BCC excisions performed before the vismodegib treatment cycle was initiated, more tumor cells were found near CD8 cells compared to samples taken while patients were on vismodegib “on”/ “off” cycles (Fig. 6D).

When patients were treated with vismodegib, there were significantly fewer CD8 T cells within a 500μm-wide area surrounding the tumor and fewer total CD8 T cells across the tumor section (Fig. 6E) compared to resections before vismodegib treatment had started. CD4 cell infiltration was also attenuated upon vismodegib treatment (Fig. S6F, G).

Taken together, our data indicate that vismodegib treatment has a previously unappreciated effect on the CD8 T cell response, inhibiting T cell migration. This effect is distinct from the direct anti-tumor effect of vismodegib on the cancer cells themselves. Thus, our work might explain why vismodegib did not have the desired efficacy across cancer types in the clinic.

## Discussion

Potent inhibitors of the Hh pathway, mainly targeting the key signal transducer SMO, have been developed and shown efficacy in BCC^31^ and SHH medulloblastoma^32^ which harbor Hh driver mutations. However, Hh inhibitors have repeatedly failed to meet primary endpoints in clinical trials for cancers where Hh signaling is aberrantly amplified (Table S1). Especially poor responses have been observed in the clinic when Hh inhibitors have been used for the treatment of gastrointestinal cancers.

Guided by the clinical data, we chose a murine model of colorectal cancer, where Hh inhibitors performed poorly. Strikingly, we observed that Hh inhibition with sonidegib (LDE225) treatment led to a doubling of the tumor burden in the animals. Of note, we have shown that sonidegib treatment also did not reduce the tumor burden in a subcutaneous KPC-derived pancreatic cancer model (2838c3) or a subcutaneous melanoma model (Yummer 1.7) (Fig. S7). This is in contrast to published work in a murine model of pancreatic ductal adenocarcinoma using the same inhibitor which resulted in a reduction of tumor burden^33^. The discrepancy in tumor response is likely due to the different model system used with regards to the tumor cell line and site of implantation.

Unlike previously published work, Hh inhibition in our model had no effect on the TME overall where tumor stroma, blood vessels and main immune subsets including macrophages and B cells remained unchanged. Previous work in murine models of breast cancer had observed a shift from Treg to Th17 cells and M2 to M1 macrophage polarization upon vismodegib treatment with no effect on tumor growth reported^34, 35^. In a hepatoma model vismodegib also led to reduced M2 polarization resulting in enhanced T cell infiltration and reduced tumor growth^36^. In our model, we did not investigate Th subsets specifically and did not observe a change in overall macrophage abundance by F4/80 staining or M1/M2 polarization using ConsensusTME.

Instead of broad changes in the TME, we find that Hh inhibition specifically abolishes cytotoxic lymphocytes infiltration into the tumor, especially the infiltration of cytotoxic CD8 T cells that are essential for tumor control. We observed a small reduction in CD4 and NK cells and it would be interesting to establish whether Hh inhibitors also affect the migration of those cells in better suited models. We further characterized the migration defect in CD8 T cells using bespoke *Smo* knockout models and adoptive transfer experiments and confirmed that the migration defect is T cell intrinsic. Migration assays and live imaging approaches revealed that CTLs lacking *Smo*, or ones treated with range of clinical and non-clinical Smo inhibitors have profoundly diminished migratory capacity. Additional work using knockout models of *Ihh* and *Gli1* as well as inhibitors and agonists of the pathway, demonstrated that CTL migration relies on non-canonical Hh signaling via Smo, independently of exogenous and endogenous Hh ligands or a Gli-mediated transcriptional program. Interestingly, the effect of Smo inhibitors on T cell migration is independent of the effect of Smo inhibitors on CTL killing, which is regulated via canonical Hh signalling via Gli1^22^. Furthermore, the reduced migratory capacity could not be attributed to altered expression of chemokine receptors on the cell surface of CTLs. Instead, we find that it is the GPCR function of Smo that regulates migration, and that *Smo* knockout CTLs have reduced levels of active, GTP-bound RhoA.

A role for the GPCR function of Smo via RhoA has been previously described in fibroblast migration^28^. In that paper, migration is linked to both G_i_ coupling and PI3K activity downstream of Smo, but independent of the Smo c-tail^28^. The c-tail of Smo is necessary for Gli activation and β-arrestin recruitment but dispensable for Gi coupling^37^. In our work, we have established a role for G_i_ coupling (PTX) and RhoA (ROCKi) in CTL migration similarly to what has been described for fibroblasts, but we have also established a role for β-arrestin recruitment (CCG). Of note, we find that CTLs with a gain-of-function *SmoM2* mutation^38^ did not exhibit improved migration (Fig. S5D). SmoM2 has been linked to G_i_ coupling in fibroblasts^39^ but it is unknown whether that is also the case for T cells. It would be interesting to determine the contribution levels of Smo versus other GPCRs towards G_i_ coupling, RhoA activation, and β-arrestin recruitment during CTL migration and explore other ways to enhance Smo-mediated T cell migration for therapy.

Our data from BCC biopsies from patients treated with vismodegib showed that treatment specifically diminished the T cell population near tumor cells. A previous report looking at five patients before and after four weeks of vismodegib treatment had suggested an increase of CD8 cells upon treatment^40^. The discrepancy may stem from the limited sample size in both cases or the differences in treatment duration. Further clinical investigations exploring T cell infiltration upon SMO inhibitor treatment on larger patient populations are warranted.

Here, we provide a comprehensive functional assessment of the anti-tumor immune response upon Hh inhibition *in vivo* and reveal a profound and selective effect on cytotoxic T cells infiltration while other immune subsets are less affected.

Our work has substantial implications for the clinic and may explain why trials of Hedgehog inhibitors in solid tumors have yielded mixed results. Furthermore, it might provide a roadmap for an improved Hh inhibitor trial design which would selectively target the tumor but spare the immune compartment.

One approach would be to investigate whether sufficient treatment breaks when patients are on SMO inhibitors would allow enhanced T cell infiltration into the tumor microenvironment. This could be extremely important when Hh inhibition is combined with immune checkpoint blockade such as anti-PD1 (Pembrolizumab). Another approach would be to selectively target Hh ligands and their processing, e.g. through Hedgehog acetyltransferase inhibitors^41, 42^, and thus avoiding inhibitory effects on CD8 T cell migration. Caveats of the two approaches might be the development of resistance during treatment breaks and differential tumor dependancy on Hh acetyltransferase activity.

Most importantly, the work uncovers Hh as a regulator of cytotoxic T cell migration into the tumor microenvironment which might inform new strategies to increase migration in T cell therapeutics.

## Materials and Methods

### Study design

The goal of this study was to determine the effects of Hh inhibitor treatment on the immune cells in the tumor microenvironment. We used small molecule antagonists and genetic knockout models of key Hh signalling components as well as *in vivo* tumor models and resected BCCs from patients treated with the Smoothened (SMO) inhibitor vismodegib. A biostatistician was consulted to determine the animal number per condition allowing the detection of the targeted effect size(s) with a probability of 0.9 when considering a family wise type I error rate of 5% for the primary endpoint(s). Where possible, we started with 5 mice per group per condition per experiment. All mice experiments were repeated at least once. More independent experiments were performed on transgenic mice where 5 littermates were not available for each cohort. Different genotypes were randomly distributed across littermates and cages. Male and female mice were used in experiments except for inhibitor studies where only female mice were used. For sonidegib treatment and adoptive transfer of CD8 cells groups were stratified depending on tumor growth. Tumors were grown for a total of 16-24 days, as indicated in respective experimental designs and rate of tumor growth, so that the tumor never exceeded 10% of the total mouse body weight. Mice with adverse health effects (e.g. ulcerations or fighting wounds) that needed to be culled before the cohort endpoint and mice without tumor growth were excluded from analysis. After data analysis, outliers were identified and excluded using the ROUT method with Q=1%. Mice were processed for endpoint analysis according to ID numbers and not treatment/genotype groups. The researcher was blinded to treatment and genotype until the data analysis was completed and retrospectively added to the samples. Different treatment/genotype groups were mixed among cages and co-housed to enable blinding and reduce housing-originating bias.

### Mice

*RAG2*KO were a generous gift from Suzanne Turner (University of Cambridge) and OTI mice were purchased from the Jackson Laboratory (*C57BL/6-Tg (TcraTcrb*)1100Mjb/j, Stock #003831). OTI *RAG2*KO mice were generated from these. *Smo*^*f/f*^ (Stock no. *Smo*^*tm2Amc*^*/J*, 004526) and *Ihh*^*f/+*^ (*Ihh*^*tm1Blan*^/J, Stock no. 024327) were purchased from The Jackson Laboratory. *GzmB*^*ERT2Cre*^*/ROSA26EYFP* mice^25^ were a generous gift from D. T. Fearon (Cold Spring Harbor Laboratory). *R26SmoM2* mice were purchased from the Jackson Laboratory (JAX stock #005130)^43^. *dLckCre* and *ROSA26tdTom* mice were a generous gift from Randall Johnson and Douglas Winton (University of Cambridge), respectively. Smo^fl/fl^ mice were crossed to *GzmB*^*ERT2Cre*^*/ROSA26tdTom* mice to generate *GzmB*^*ERT2Cre*^*/ROSAtdTom/Smo*^*fl/fl*^ mice. *Ihh*^*fl/fl*^ mice were crossed onto *dLckCre/ ROSA26tdTom* mice. *Gli1-eGFP* mice^44^ were a generous gift from Alexandra Joyner (Sloan Kettering Institute). All strains were backcrossed onto the C57BL/6J background (Charles River, UK) for more than 10 generations.

Mice were genotyped using Transnetyx, maintained at the CRUK Cambridge Institute-University of Cambridge and used after 6 weeks of age. All housing and procedures were performed in strict accordance with the United Kingdom Home Office Regulations.

### Tumor models

C57BL/6J or *Rag2*KO or *GzmB-Cre*^*ERT2*^*Smo*^*+/fl, fl/fl*^ mice were injected subcutaneously with tumor cells on d0. MC38 (Kerafast #ENH204-FP) or MC38-OVA cells were injected at 0.5 × 10^6^ in PBS. MC38-OVA were generated by retroviral transduction of MC38 cells with HEK293T supernatant transfected with a pMig-cytoOVA-IRES-tdTomato plasmid. For *GzmB-Cre*^*ERT2*^*Smo*^*+/fl, fl/fl*^ adoptive transfer, mice were treated with 75mg/kg tamoxifen (Merck #T5648) diluted in corn oil (Merck #C8267) one day before tumor implantation (-d1) and then again on d1, d3 and d5. For KPCY line 2838c3^45^ (Kerafast #EUP013-FP) cells were co-injected 1:1 with Matrigel (Cultrex, Bio-techne #3433-010-R1). YUMMER 1.7 melanoma cells were a generous gift from Marcus Bosenberg^46^ and were injected at 0.5 × 10^6^ in PBS. All tumor lines tested negative for mouse pathogens (M1 PCR panel, Surrey Diagnostics). Tumors were grown for a total of 16-24 days, as indicated in respective experimental designs and rate of tumor growth, so that the tumor never exceeded 10% of the total mouse body weight. Mice were treated with sonidegib (sonidegib diphosphate salt (LC labs #S-4699) dissolved in polyethylene glycol 400 (Sigma #91893) and 5% dextrose (Sigma #G8270) in water, 75:25 v/v) or carrier control and administered by oral gavage at 20mg/kg daily for up to 12 days. This dose was chosen for sustained Gli1 inhibition^14^. For adoptive transfer experiments of cytotoxic CD8 T cells, mice were first stratified into two equal groups according to tumor size, and then 1 × 10^6^ cells were injected intravenously on d12. Tumor size was assessed by caliper measurements and tumor volume was calculated using the formula:

Tumoursize(mm)=π/6×(biggerdimension×smallerdimension×smallerdimension)

### Tissue culture and cell lines

Murine CD8 T cells were cultured at 1x10^6^ cells/ml in complete RPMI media: RPMI 1640 Medium (Thermo #21875034) supplemented with 10% heat-inactivated, batch-tested FCS (Biosera #1001), 50µM β-Mercaptoethanol (50mM, Thermo #31350010), 100U/ml Penicillin/Streptomycin (10,000 U/ml, Thermo #15140122), 1µM Sodium Pyruvate (Gibco, #11360039), 10µM HEPES (Sigma #H0887) and 10ng/ml murine IL-2 (Peprotech #212-12).

HEK293T, MC38, MC38-tdTom, MC38-Cyto-OVA-tdTom and KPCY (clone 2838c3) cells were cultured in 10% heat-inactivated FCS DMEM (Thermo #41966029). YUMMER 1.7-tdTom were cultured in 10% FCS Ham’s F-12 Nutrient Mix (Thermo #21765029) supplemented with NEAA (Thermo #11140035).

All cells were grown in a humidified incubator at 37°C and 5% CO2. Cell lines were STR profiled and all tested mycoplasma negative (PhoenixDx mycoplasma qPCR kit, Procomcure #PCCSKU15209).

### Murine CD8 cell isolation and expansion and *in vitro* treatment

Total murine CD8 T cells were isolated using MACS (negative selection, Miltenyi Biotec #130-104-075) according to the manufacturer’s instructions. The purity of the sorted populations was above 95%. CD8 T cells were stimulated with platebound anti-CD3ε (1µg/ml, eBioscience #16-0033-86) and anti-CD28 (2µg/ml, eBioscience #16-0281-86) antibody. Alternatively, cell suspensions from spleen and lymph node from OTI mice were stimulated with 10nM Ova257-264 peptide (SIINFEKL, Cambridge Bioscience #60193-5-ANA). T cells were stimulated for 48hrs and grown for up to 11 days. Cells were considered cytotoxic (CTL) between d6-d8 and used at that time for adoptive transfers, killing assays and migration assays. Cells were restimulated on d10 with 1μg/ml plate-bound anti-CD3e (eBio500A2 (500A2), functional grade, eBioscience #16-0033-86) for creation of cell pellets for RNA or protein extraction.

For CTL generation from *GzmBCre*^*ERT2*^*/ROSAtdTom/Smo*^*+/fl*,*fl/fl*^ mice *in vitro*, cells were incubated with 4-hydroxytamoxifen (4-OHT, Bio-techne #341210) diluted in DMSO (Merck #D12345) in order to activate the CreERT2 recombinase. Cells were treated at a concentration of 300nM and fresh 4-OHT was added daily for the first five days of culture. Control-treated cells received DMSO in complete RPMI.

### Human CD8 cell isolation and expansion and *in vitro* treatment

PBMCs were obtained from peripheral blood samples. Samples were taken from sporadic BCC patients enrolled in the ALF research study (Research into Altered Lymphocyte Function in Health and Disease, IRAS 220302, 17/YH/0304). Buffy coats were obtained from NHS Blood and Transplant under the ALF ethics.

To isolate PBMCs, total blood products were diluted in 2% FCS PBS and laid over Ficoll density gradient medium (Merck #17-1440-02) in SepMate 50ml tubes (Stemcell Technologies, #85460). Subsequently, CD8 cells were isolated using the MojoSort™ Human CD8 T Cell Isolation Kit (Biolegend # 480129) according to the manufacturer’s instructions. Cells were stimulated for 48h with ImmunoCult™ Human CD3/CD28/CD2 T Cell Activator (25 µL/mL/1×10^6^ CD8 cells; Stemcell Technologies, #10970). CD8 T cells were cultured in Immunocult (Stemcell Technologies #10981) supplemented with 100 U/ml human IL-2 (Miltenyi Biotec #130-097-746) and 100 U/ml Penicillin/Streptomycin (Gibco).

### CD8 T cell migration assay

Incucyte chemotaxis and reservoir plates were used (Clear View 96-well Chemotaxis plate for cell migration and reservoirs, Sartorius #4582 and #4600) and the following method was adapted from the Sartorius “Incucyte® Chemotaxis Cell Migration Assay” protocol. Insert wells were coated with 0.5% Matrigel (Cultrex, Bio-techne #3433-010-R1) in PBS.

CTLs were pre-treated with small molecules or carrier controls at the indicated concentrations (Table S3) for 1hr in T cell media before the assay. 60μl of cell suspension containing small molecules were plated in each well of the insert plate containing 7000 CTLs/well. The reservoir plate was filled with pre-warmed media containing 200nM Cxcl10 and 125nM Cxcl11 or seeded tumor cells. The insert plate was placed on top of the reservoir plate and left for 6-8hrs at 37°C and 5% CO_2_. Afterwards, the insert plate was discarded and cells from the reservoir plate were aspirated, stained, and enumerated by flow cytometry.

Murine CTLs were used between d6-8 and were resuspended in 5% FCS complete RPMI on the top well. The reservoir plate was filled with 30% FCS complete RPMI containing 200nM Cxcl10 and 125nM Cxcl11 (Peprotech #250-16-250 and #250-29-250, respectively). Alternatively, the reservoir was seeded overnight with 6,000 MC38-OVA cells in 10% FCS DMEM/well. Just before the insert plate was added, the DMEM medium was aspirated and replaced by 30% FCS complete RPMI.

Human CTLs were used between d10-12 and were resuspended in Immunocult supplemented with 100 U/ml human IL-2. The reservoir plate was filled with the same media supplemented with 200nM CXCL10 and 125nM CXCL11 (Peprotech #300-12-100 and #300-46-100, respectively).

### RNA isolation

To validate the targeting of the Hh pathway by sonidegib in the tumor mouse cohorts, we collected a small piece of the gut, approximately 0.5 cm of the proximal duodenum. The tissue was washed briefly in PBS and flash frozen in Precellys lysis tubes (Precellys #432-3751) and subsequently homogenised in a Precellys Evolution Touch Homogeniser (3 cycles,15 sec, 6000 rpm, 4°C). RNA was extracted using the PureLink RNA Mini kit (Thermo #12183025) according to manufacturer’s instructions and RNA concentration was assessed by a Nanodrop spectrophotometer.

### qRT-PCR

Reactions for qRT-PCR were set up in 384 well plate format using the One-Step qRT-PCR Kit (Thermo Fisher SuperScript III Platinum #11732088) and Taqman probes (Thermo Fisher, Table S4), according to manufacturers’ instructions. Each sample was run in triplicate with *Tbp* serving as housekeeping gene. Reverse transcription thermal cycling was carried out on a QuantStudio 6 Flex Real-Time PCR System (Thermo Fisher). Gene expression was calculated with the ΔCt method^47^. The cycle threshold (Ct) value from the gene of interest was subtracted from the housekeeping gene and transformed with a factor of 2^(-ΔCt) to give the fold expression relative to the housekeeping gene.

### Bulk RNA Seq

CD8 T cells were purified from spleens of *GzmBCreERT2/Rosa26eYFP/Smo* WT, HET and KO and stimulated for two days with plate-bound antibodies against CD3ε and CD28 in complete T cell media containing 300nM 4-OH-Tamoxifen (4-OHT) in presence of 100IU/ml IL-2. On d5, live CD8 eYFP positive and negative cells were FACS sorted, and cells were maintained in complete T cell media enriched with 50IU/ml IL-2. On d6, cells were harvested, and RNA was extracted using the miRNeasy mini kit (Qiagen #217004). RNA quality was assessed by 2100 Bioanalyzer Instrument (Agilent) using RNA 6000 Nano Kit (Agilent #5067-1511) and RNA concentrations were quantified using the Qubit RNA BR Assay kit (Thermo #10210). Libraries were generated using the TruSeq stranded mRNA kit (Illumina) and sequenced using single-read sequencing with the HiSeq4000 platform (Illumina). Reads were aligned to the mouse reference genome GRCm38132 using STAR v2.7.2b133 with default parameters and quality control was assessed using FASTQC v0.11.8 and Picard v2.9.5. Counts of reads against genes were generated using featureCounts from the Subread package v1.5.2134 and read counts were normalised and tested for differential gene expression using the DESeq2 package v1.5.2135.

### ConsensusTME

ConsensusTME is a published computational method that provides cell-type population estimates from bulk RNA-seq data. It integrates experimentally validated and de novo gene signatures from seven other TME cell estimation methods to generate the consensus gene superset for each cell type.

A small part of the tumor mass resected from the mouse cancer models was flash frozen in liquid nitrogen immediately after harvest. Samples were processed as previously described^24^. Gene expression was assessed by RNA-sequencing on a NovaSeq 6000 instrument. Reads were trimmed using Trimmomatic (v0.40)^48^ and alignment was performed using HiSat2 (v.2.2.1)^49^. ConsensusTME (v0.0.1.9000)^24^ was run with mouse gene sets derived from immunological genome project^50^ data to estimate immune cell abundance from mouse RNA-seq data.

### RhoA G-LISA

The G-LISA Rho activation assays (Cytoskeleton #BK-124) are colorimetric, ELISA based Rho activation assays which measure the level of GTP-loaded RhoA in cells. Briefly, 20×10_6 of d7 CD8+ CTLs expanded *in vitro* as above were stimulated with 1μM LPA for 5 minutes. Cells were subsequently washed and lysed in the buffer provided by the BK-124 together with protease inhibitors (Fisher #A32955). Cells were spun and protein concentration in the supernatant was quantified by Direct Detect (Millipore) and equalised so that the protein concentration was the same among all samples. Equalised supernatants were assayed according to manufacturer’s instructions and measured with absorbance set at 490nm using a CLARIOstar microplate reader (BMG Labtech).

### Western Blot

#### Protein Immunoblot

Cells were harvested at 4°C, washed twice in ice-cold PBS and lysed in ice-cold RIPA buffer (150mM NaCl, 50mM Tris pH 7.4, 1mM MgCl_2_, 2% NP40, 0.25% Na deoxycholate, 1mM DTT) with protease inhibitor cocktail (Pierce #88669). Protein concentration was determined with the Direct Detect spectrometer (Merck Millipore). Lysates were run on a NuPAGE 4-12% gradient Bis/Tris Acrylamide gel (Thermo Fisher #NP0335BOX). Protein immunoblotting was performed using wet transfer onto 0.45 µm nitrocellulose membrane (Thermo Fischer #LC2001) using the BioRad mini-transblot system. Membranes were blocked with 5% (w/v) non-fat dry milk (Marvel Original, Dried Skimmed Milk) in TBST and incubated with primary and secondary antibodies shown in Table S5. Membranes were incubated with stripping buffer (Restore PLUS Western Blot Stripping Buffer, Thermo Fisher #46430) between probing. Samples were visualised with SuperSignal West Pico Plus Chemiluminescent Substrate (Thermo Fisher #34580). Whole scans shown in Data File S3.

#### Flow Cytometry

Cells were stained with fixable viability dye eFluor780 (eBioscience #65-0865-18), washed, and incubated with Fc block (1:100; Biolegend TruStain fcX #101320) and fluorophore-conjugated antibodies at the appropriate concentrations (Table S6) for 20min at 4°C. Flow cytometric analyses were conducted on a BD Fortessa or Symphony cell analyser, and data was analysed with FlowJo software (Tree Star Inc., v10.10). Gating strategy is shown in Fig. S1D.

#### Immunohistochemistry

Tissues were fixed in neutral buffered formalin (Sigma #HT5014) for 24hrs and then in 70% EtOH for 24hrs. Following fixation, the tissues were embedded in paraffin and sectioned at 3 μm in a Leica microtome. H&E and antibody staining was run on the Leica Bond-III platform according to CRUK-CI Histopathology facility protocols. The sodium citrate (Leica’s Epitope Retrieval Solution 1 #AR9961) and Tris EDTA (Leica’s Epitope Retrieval Solution 2 #AR9640) pre-treatments were performed at 100°C. The ancillary reagents are as follows: protein block from (Dako #X090930-2), anti-rat secondary (1:250, Bethyl Laboratories #A110-322A) and DAB Enhancer (Leica #AR9432) was applied to all antibodies. Antibodies and conditions are listed in Table S7.

#### Immunohistochemistry analysis

To quantify CD8 infiltration into mouse tumors, serial tumor sections were stained with anti-mouse CD3 and CD8 as described above. Each tumor was subdivided in 100 μm-wide concentric rings and CD3+CD8+ double positive cells were enumerated per ring (HALO v3.6.4134.137).

#### Live imaging

35mm Glass bottom dishes (MatTek #P35G-1.0-14-C) were coated with ICAM-1 (at 4°C, overnight at 1.25 μg/ml in PBS, BioTechne #796-IC-050). CTLs on days 6-8 after stimulation *in vitro* from *Smo* WT and KO or CTLs from *Rag1*KO OT-I mice were stained with CFSE (1:10,000 in PBS, Thermo #C34554) for 1h at 37°C. Where indicated, cells were pre-treated with cyclopamine overnight. CFSE-labelled CD8 T cells were washed and resuspended at 278,000 cells/ml in complete T cell media containing collagen (0.3 mg/ml PureCol Type I Bovine Collagen, Advanced Biomatrix #5005-100). Experimental setup and analysis was informed by de Boer et al, 2023^51^ and Roy et al, 2018^52^. Cell suspensions were added to the coated dishes and allowed to settle for 15 min in a humified chamber of an Andor Dragonfly 500 confocal spinning disc microscope (Oxford Instruments). Cells were imaged using the 40x oil immersion objective. Each picture is a composite of XY: 4×2 tiles, Z: 25 µm (2µm step size) and T: 20 min (20 sec interval).

#### Image acquisition and analysis

Images were processed using Imaris software (Bitplane/Oxford Instruments). ‘Spots’ function was used to detect cells and the ‘tracking module’ function was used to map cell tracks. Mean speed, length and straightness of tracks were analysed for fully attached cells with tracks over 10 min long. 90-150 cell tracks were analysed per sample. The xy migration plots were based on cell tracks of speed >0.01 μm/sec using the spot function ‘plot all tracks with common origin’ from the Biological Imaging Development CoLab (BIDC) at UCSF Parnassus Heights.

#### Clinical Trial Database

Results of published Hh inhibitor clinical trials on ClinicalTrials.gov were compiled and ordered according to cancer type and colour-coded according to complete (CR) or partial (PR) objective response rates (ORRs) in patients (blue – positive effect; red – negative effect) (Table S1 and Data File S1). Search terms used were ‘Sonidegib’, ‘Vismodegib’, ‘Arsenic Trioxide’ and ‘Itraconazole’. When both phases I and II are listed, the response rates and number of participants from phase II are reported. Number of patients exclusively includes number of patients whose response to treatment was evaluated (not number initially enrolled, if those differ from final number). Trials published up to April 2024 were included in the compiled data.

### Statistical analysis

For the statistical analysis of two groups, a two-sample unpaired t-test was performed. For analysis of multiple groups, one-way analysis of variance (ANOVA) with Tukey’s or multiple comparisons test was applied. For multiple testing, a two-way ANOVA with Sidak’s multiple comparisons test was applied. Family-wise significance and confidence level was set at P < 0.05. If not otherwise described, statistical examinations were carried out using GraphPad Prism (v.10.2.0). Additional information on the study design, the number of replicates and the statistical tests used is provided in the figure legends. Graphs and illustrations were created using GraphPad Prism and Affinity Designer (v.2.0.0).

## Supplementary Material

Supplementary Materials

## Data Availability

All data needed to support the conclusions of the paper are present in the paper or the Supplementary Materials. Bulk RNA-Seq data used for ConsensusTME can be accessed in Data File S2. Whole protein immunoblot scans can be viewed in Data File S3. Raw data can be found in Data File S4.

## One Sentence Summary

Smoothened inhibitors decrease CD8 T cell migration into murine and human tumors.
