# Supplementary material for: Non-canonical Hh signalling through Smoothened controls cytotoxic T cell migration in the tumor microenvironment
Source: Sci Immunol. Author manuscript; Available in PMC 2026 Jun 2. (PMC7619125; doi:10.1126/sciimmunol.adr3127)
Supplement: Supplementary Materials [file EMS212618-supplement-Supplementary_Materials.pdf]

LIST OF SUPPLEMENTARY MATERIALS

Table S1: Clinical trials of Hedgehog inhibitors in cancer patients have shown low efficacy in non-Hh-driven malignancies.

Figure S1: Sonidegib treatment diminishes the lymphoid compartment in the tumor.

Figure S2: Infiltration analysis in MC38 tumors treated with sonidegib.

Figure S3: Genetic loss of *Smo* but not *Ihh* or *Gli1* in cytotoxic CD8 cells does affect tumor infiltration and growth.

Figure S4: Genetic loss of *Smo* in cytotoxic CD8 cells does not affect general cytokine production profiles or response to tumor chemoattractants via the Cxcr3-Cxcl9/10/11 axis.

Figure S5: *Smo* deletion does not affect steady state levels of RhoA, Rac1, and Cdc42 and *SmoM2* mutation has no effect in T cell migration *in vitro*.

Figure S6: Image analysis of BCC biopsies shows diminished CD4 T cell infiltration upon vismodegib treatment.

Figure S7: Sonidegib treatment fails to reduce the tumor burden in murine models of pancreatic cancer or melanoma.

Table S2: Clinical information of BCC biopsies.

Table S3: Small molecules used for migration assays.

Table S4: Probes used for qRT-PCR.

Table S5: Antibodies used for protein immunoblot.

Table S6: Antibodies used for flow cytometry.

Table S7: Antibodies used for immunohistochemistry.

Data File S1: Extended table of clinical trials of Hedgehog inhibitors in cancer patients.

Data File S2: Bulk RNA-Seq data used for ConsensusTME.

Data File S3: Protein immunoblot scans.

Data File S4: Raw data file.

SUPPLEMENTAL MATERIAL

Cancer	NCT	Drug	ORR	
Basal Cell Carcinoma (laBCC)	NCT02667574	V	0.71	
	NCT01367665	V	0.69	
	NCT00833417	V	0.60	
	NCT00607724	V	0.60	
	NCT01815840	V	0.58	
	NCT01327053	S	0.51	
Basal Cell Carcinoma (mBCC)	NCT00607724	V	0.50	
	NCT00833417	V	0.49	
	NCT01367665	V	0.37	
	NCT01327053	S	0.13	
	NCT02690948	V+P	-0.15	
Medulloblastoma	NCT01601184	V+Ch	0.20	adult - MB with Hh signature
	NCT01125800	S	0.19	adult - all responders are Hh-positive
	NCT00939484	V	0.15	adult - MB with Hh signature
	NCT01239316	V	0.08	paediatric - MB with Hh signature
	NCT01125800	S	0.05	paediatric - all responders are Hh-positive
	NCT01239316	V	0.00	paediatric - MB without Hh signature
	NCT00939484	V	0.00	adult - MB without Hh signature
	NCT00939484	V	0.00	adult - MB with inconclusive Hh
Lung Cancer	NCT00887159	V+Ch	0.08	small-cell lung cancer; no significant difference compared to chemotherapy alone
Prostate cancer	NCT02111187	S	0.00	60% higher PSA levels in the Sonidegib arm relative to Ctrl
	NCT02115828	V	0.00	100% PSA Increase as best response - metastatic prostate cancer
Glioblastoma	NCT00980343	V	0.00	
Sarcoma	NCT01267955	V	0.00	chondrosarcoma - overexpression of the Hh ligand was observed in 13 cases (65%)
	NCT01154452	V+Gsi	0.00	liposarcoma, clear cell sarcoma, chondrosarcoma, alveolar soft part sarcoma or chordoma
Lymphoma	NCT01944943	V	0.04	
Leukemia (AML)	NCT01546038	G+Ch	0.16	newly diagnosed acute myeloid leukemia
	NCT01826214	S	0.01	relapsed/refractory acute leukemia
Pancreatic cancer	NCT01195415	V+Ch	0.00	
	NCT01088815	V+Ch	0.00	
	NCT01064622	V+Ch	-0.05	
Stomach cancer	NCT00982592	V+Ch	-0.07	advanced gastric and gastroesophageal junction (GEJ) adenocarcinoma
Colorectal cancer	NCT00636610	V+Ch+B	-0.05	

Table S1: Clinical trials of Hedgehog inhibitors in cancer patients have shown low efficacy in non-Hh-driven malignancies.

Clinical trials using Hh inhibitors in cancers with driver mutations in the Hh pathway (laBCC, mBCC, a subset of medulloblastoma patients) and cancers with an amplified Hh signature (lung, prostate, sarcoma, leukemia, pancreatic, stomach and colorectal). Overall Response Rate (ORR) shows the percentage of complete responders or partial responders. When indicated, Hh inhibitors were administered in addition to the current gold standard of treatment. In those cases, the ORR represents the difference between the response rate of the standard treatment alone versus standard treatment in combination with Hh inhibitor. Abbreviations: BCC: Basal Cell Carcinoma, laBCC: locally advanced BCC, mBCC: metastatic BCC, NCT: ClinicalTrials.gov identifier, V: vismodegib,

S: sonidegib, P: pembrolizumab, Ch: chemotherapeutic agents (respective standard of care), Gsi: Gamma-secretase Inhibitor RO4929097, G: glasdegib, B: bevacizumab. Detailed database is provided as a **Data File S1**.

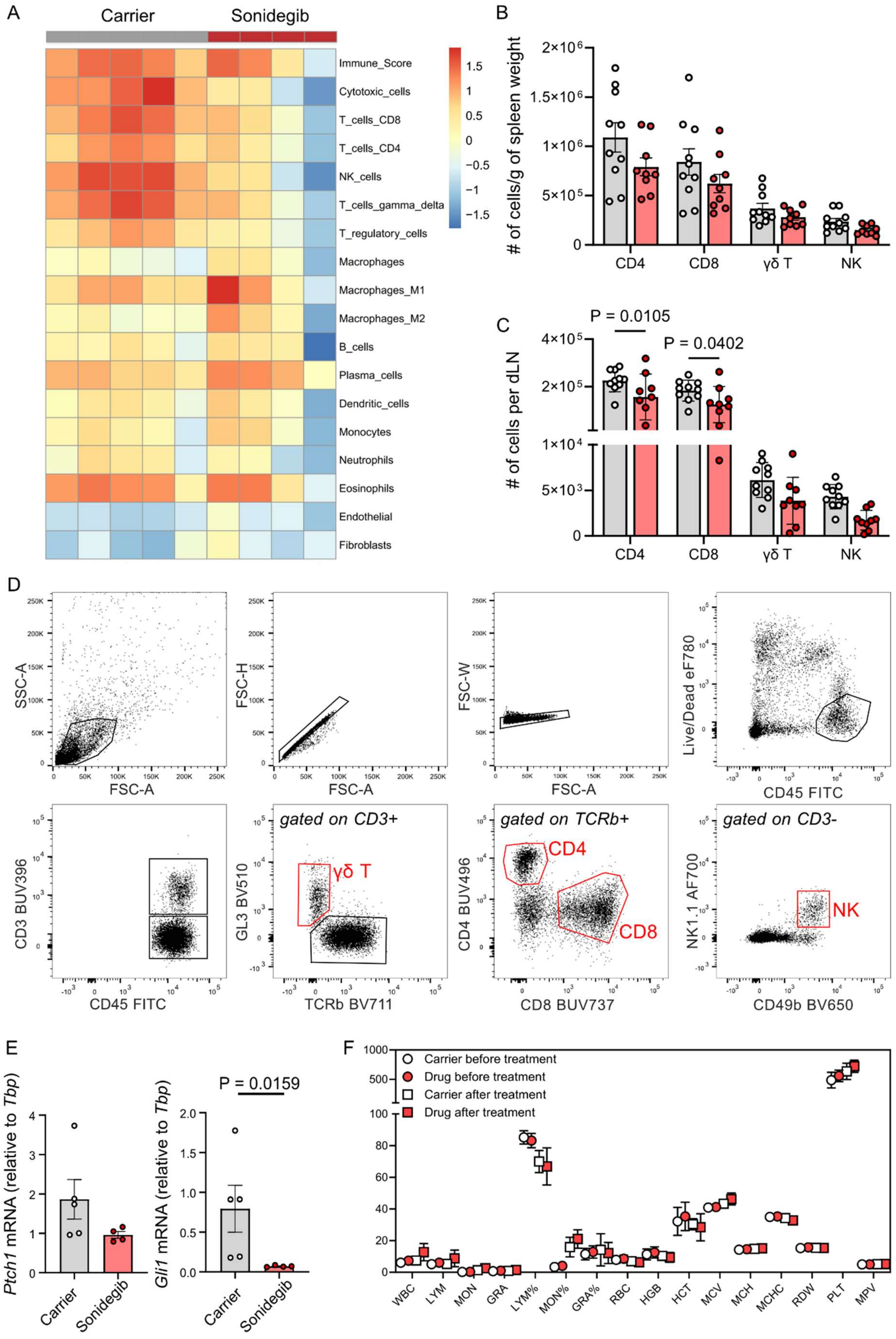
A
Carrier
Sonidegib
Immune_Score
Cytotoxic_cells
T_cells_CD8
T_cells_CD4
NK_cells
T_cells_gamma_delta
T_regulatory_cells
Macrophages
Macrophages_M1
Macrophages_M2
B_cells
Plasma_cells
Dendritic_cells
Monocytes
Neutrophils
Eosinophils
Endothelial
Fibroblasts
1.5
1
0.5
0
−0.5
−1
−1.5
B
# of cells/g of spleen weight
CD4
CD8
γδ T
NK
C
P = 0.0105
P = 0.0402
# of cells per dLN
CD4
CD8
γδ T
NK
D
SSC-A
FSC-A
FSC-H
FSC-A
FSC-W
FSC-A
Live/Dead eF780
CD45 FITC
CD3 BUV396
CD45 FITC
gated on CD3+
GL3 BV510
γδ T
TCRb BV711
gated on TCRb+
CD4 BUV496
CD4
CD8
CD8 BUV737
gated on CD3-
NK1.1 AF700
NK
CD49b BV650
E
Ptch1 mRNA (relative to Tbp)
Carrier
Sonidegib
P = 0.0159
Gli1 mRNA (relative to Tbp)
Carrier
Sonidegib
F
Carrier before treatment
Drug before treatment
Carrier after treatment
Drug after treatment
WBC
LYM
MON
GRA
LYM%
MON%
GRA%
RBC
HGB
HCT
MCV
MCH
MCHC
RDW
PLT
MPV

Figure S1: Sonidegib treatment diminishes the lymphoid compartment in the tumor.

(A) Bulk RNA-Seq data of MC38 tumors from mice treated with either carrier or sonidegib analysed by Consensus-TME. n=5 for carrier-treated tumors, n=4 for sonidegib-treated tumors, one independent experiment.

(B) Numbers of CD4+, CD8+, gammadelta T cells and NK cells in the spleens of MC38 tumor-bearing mice measured by flow cytometry and shown per gram of spleen weight. n=10 for carrier-treated mice, n=9 for sonidegib-treated mice, two independent experiments, two-way ANOVA, mean ± SEM.

(C) Numbers of CD4+, CD8+, gamma delta T cells and NK cells in the tumor-draining lymph nodes of MC38 tumor-bearing mice measured by flow cytometry. n=10 for carrier-treated mice, n=9 for sonidegib-treated mice, two independent experiments, two-way ANOVA, mean ± SEM.

(D) Representative gating strategy for tumor cell populations shown in **Fig. 1H**. Doublets (based on height and width) and dead cells were excluded from the analysis.

(E) mRNA levels of *Ptch1* and *Gli1* in the skin proximal to the tumor site in the same animals as **Fig. 1C**. One independent experiment, n=5 for carrier-treated mice, n=4 for sonidegib-treated mice, unpaired t-test, mean ± SEM.

(F) Blood counts from mice treated with carrier or sonidegib shown in **Fig. 1** were assessed, before (d11) and after treatment (d24), n=5 for carrier-treated tumors, n=5 for sonidegib-treated tumors, one independent experiment, mean ± SD.

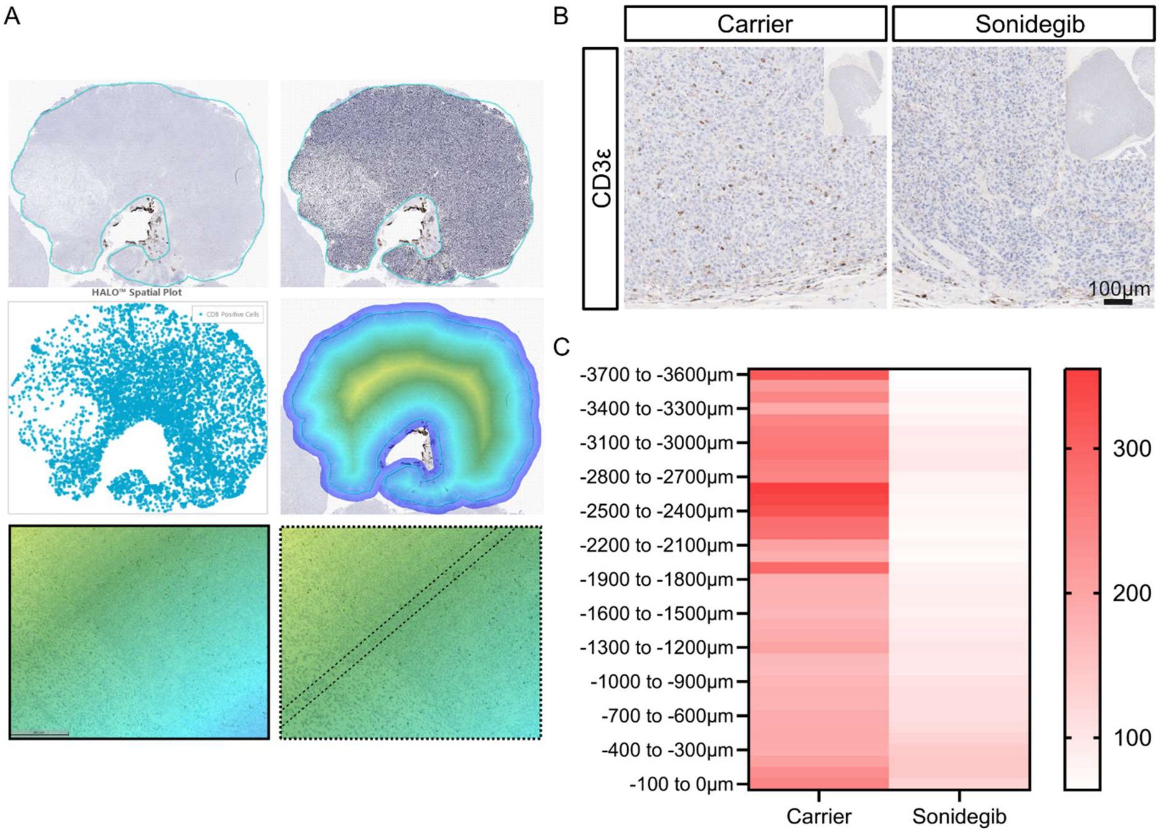

Figure S2: **Infiltration analysis in MC38 tumors treated with sonidegib.**

(A) Workflow of T cell infiltration analysis using Halo software. First mask was applied to outline the tumor margins and a second mask to indicate CD3/CD8 double positive cells. Subsequently, the tumor was subdivided in 100 µm concentric zones starting from the tumor border towards the centre of the tumor. Such analysis was performed in **Fig. 2C** and **Fig. S5C, 7G, 7N**.

(B) Representative paraffin sections of MC38 tumors from mice treated either with carrier control or sonidegib and stained with anti-mouse CD3ε antibodies. The whole tumor is shown in top right insert panels.

(C) Numbers of CD3+ cells/ mm^2 in 100µm-wide zones from the tumor surface (-100 to 0 µm) to the tumor centre (-3700 to -3600 µm) as quantified by HALO analysis (workflow shown in (**A**). Mean is shown. n=10 for carrier-treated and n=8 for sonidegib-treated mice, two independent experiments.

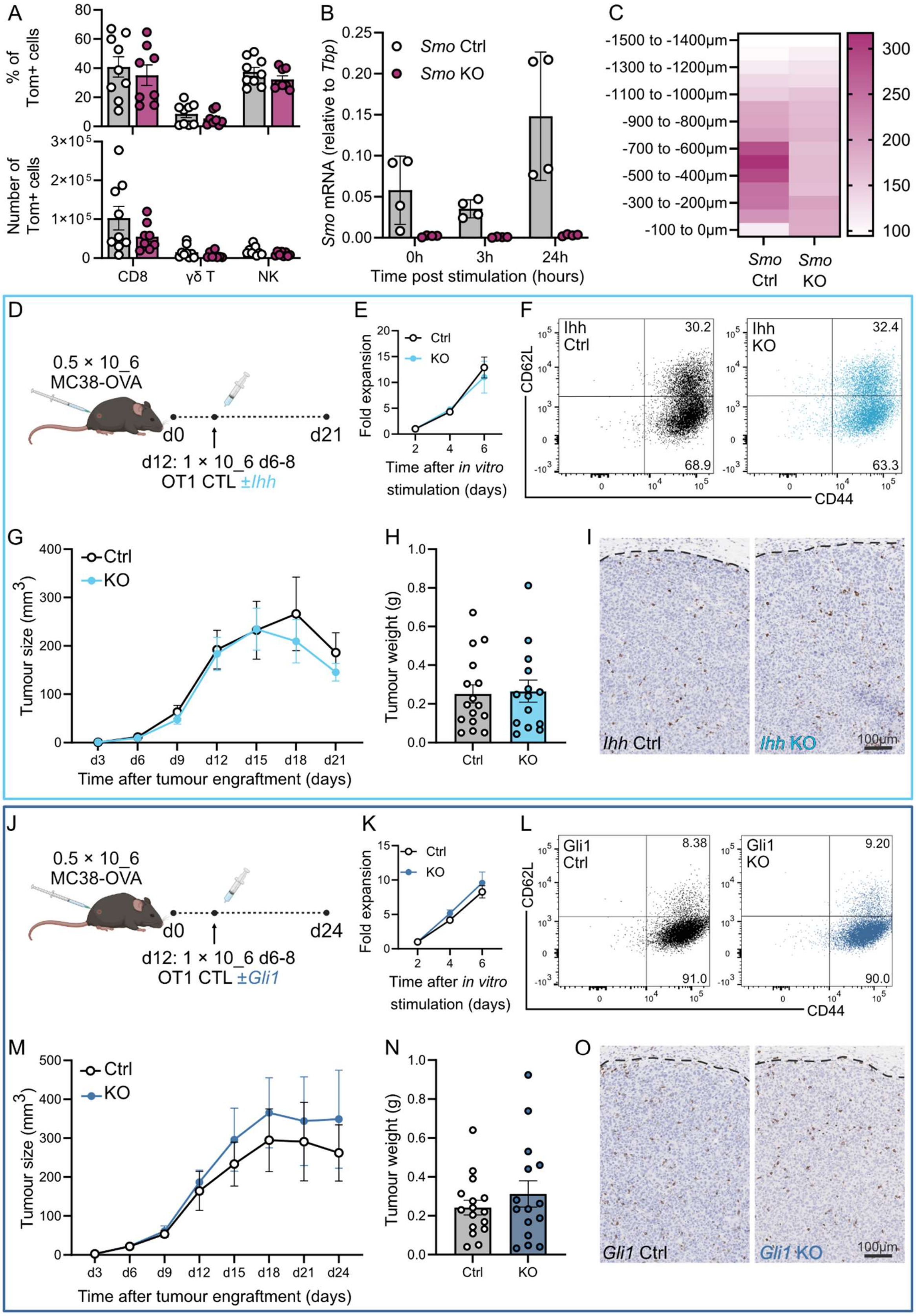
A
% of Tom+ cells
Number of Tom+ cells
CD8
γδ T
NK
B
Smo Ctrl
Smo KO
Smo mRNA (relative to Tbp)
0h
3h
24h
Time post stimulation (hours)
C
-1500 to -1400μm
-1300 to -1200μm
-1100 to -1000μm
-900 to -800μm
-700 to -600μm
-500 to -400μm
-300 to -200μm
-100 to 0μm
Smo Ctrl
Smo KO
D
0.5 × 10_6
MC38-OVA
d0
d21
d12: 1 × 10_6 d6-8
OT1 CTL ±Ihh
E
Ctrl
KO
Fold expansion
Time after in vitro stimulation (days)
F
Ihh Ctrl
Ihh KO
30.2
68.9
32.4
63.3
CD62L
CD44
G
Ctrl
KO
Tumour size (mm3)
Time after tumour engraftment (days)
H
Tumour weight (g)
Ctrl
KO
I
Ihh Ctrl
Ihh KO
100μm
J
0.5 × 10_6
MC38-OVA
d0
d24
d12: 1 × 10_6 d6-8
OT1 CTL ±Gli1
K
Ctrl
KO
Fold expansion
Time after in vitro stimulation (days)
L
Gli1 Ctrl
Gli1 KO
8.38
91.0
9.20
90.0
CD62L
CD44
M
Ctrl
KO
Tumour size (mm3)
Time after tumour engraftment (days)
N
Tumour weight (g)
Ctrl
KO
O
Gli1 Ctrl
Gli1 KO
100μm

Figure S3: **Genetic loss of *Smo* but not *Ihh* or *Gli1* in cytotoxic CD8 T cells does affect tumor infiltration and growth.**

(A) *GzmB-ERT2Cre/ROSAtdTom/Smo*$^{fl/fl}$ (KO) or *Smo*$^{fl/+}$ (Ctrl) animals were injected with 0.5 × 10^6 MC38 cells subcutaneously on d0. Mice were treated with tamoxifen via intraperitoneal injections (75mg/kg) on -d1, d1, d3 and d5. Four independent experiments, n=9 for *Smo*$^{fl/+}$ (Ctrl), n=8 for *Smo*$^{fl/fl}$ (KO). Percentage (top panel) and number (bottom panel) of immune subsets in the tumor microenvironment in which *Smo* has been excised after tamoxifen administration. Cell numbers are normalised to tumor weight.

(B) Expression of *Smo* mRNA in CD8 T cells isolated from *GzmB-ERT2Cre/ROSAtdTom/Smo*$^{fl/fl}$ (KO) or *Smo*$^{fl/+}$ (Ctrl) animals after *ex vivo* expansion and restimulation on d10 at indicated timepoints. OHT was added to the cultures for the first 5 days and *Tbp* was used as a housekeeping gene. Similar results were obtained when *CD3e* was used as a reference gene. n=4 for *Smo*$^{fl/+}$ (Ctrl), n=4 for *Smo*$^{fl/fl}$ (KO), two independent experiments.

(C) Quantification of T cell infiltration shown in **Fig. 3J** by HALO analysis. Two independent experiments, n=4 for *Smo*$^{fl/+}$ (Ctrl), n=10 for *Smo*$^{fl/fl}$ (KO), mean is shown.

(D) Experimental Design. *Rag2*KO animals were subcutaneously injected with 0.5 × 10^6 MC38-OVA cells on d0. On d12, mice were stratified into two equal groups according to tumor size. In parallel, single cell suspensions from spleens and lymph nodes of *dLck-Cre/Ihh*$^{fl/fl}$ (KO) or *Ihh*$^{fl/+}$ (Ctrl) OTI mice were stimulated *in vitro* with OVA peptide for 48hrs and subsequently cultured for 6-8 days.

(E) Fold expansion of CD8 T cells during *in vitro* culture.

(F) Representative graphs of CD62L/CD44 expression of CTLs on d7 used for the adoptive transfers (**G**, **H** of this figure).

(G) Tumor dimensions were determined by caliper measurements. n=16 for *Ihh*$^{fl/+}$ (Ctrl), n=14 for *Ihh*$^{fl/fl}$ (KO), two independent experiments, ordinary two-way ANOVA with Geisser-Greenhouse correction, mean ± SEM.

(H) Tumor weight at endpoint (d21) from (**G**), unpaired Mann-Whitney test, mean ± SEM.

(I) Representative paraffin sections from MC38-OVA tumors shown in (**G**, **H**) and stained with anti-mouse CD8α antibodies. Dotted line indicates tumor margins.

(J) Experimental Design. *Rag2*KO animals were subcutaneously injected with 0.5×10^6 MC38-OVA cells on d0. On d12, mice were stratified into two equal groups according to tumor size. In parallel, single cell suspensions from spleens and lymph nodes of *Gli1*$^{eGFP/eGFP}$ (KO) or *Gli1*$^{+/+}$ (Ctrl) OTI mice were stimulated *in vitro* with OVA peptide for 48hrs and subsequently cultured for 6-8 days.

(K) Fold expansion of CD8 cells during *in vitro* culture.

(L) Representative graphs of CD62L/CD44 expression of CTLs on d7 used for the adoptive transfers (**M**, **N** of this figure).

(M) Tumor dimensions were determined by caliper measurements. n=12 for *Gli1*$^{+/+}$ (Ctrl), n=11 for *Gli1*$^{eGFP/eGFP}$ (KO), three independent experiments, ordinary two-way ANOVA with Geisser-Greenhouse correction, mean ± SEM.

(N) Tumor weight at endpoint (d21) from (**M**), unpaired Mann-Whitney test, mean ± SEM.

(O) Representative paraffin sections from MC38-OVA tumors shown in (**M**, **N**) and stained with anti-mouse CD8α antibodies. Dotted line indicates tumor margins.

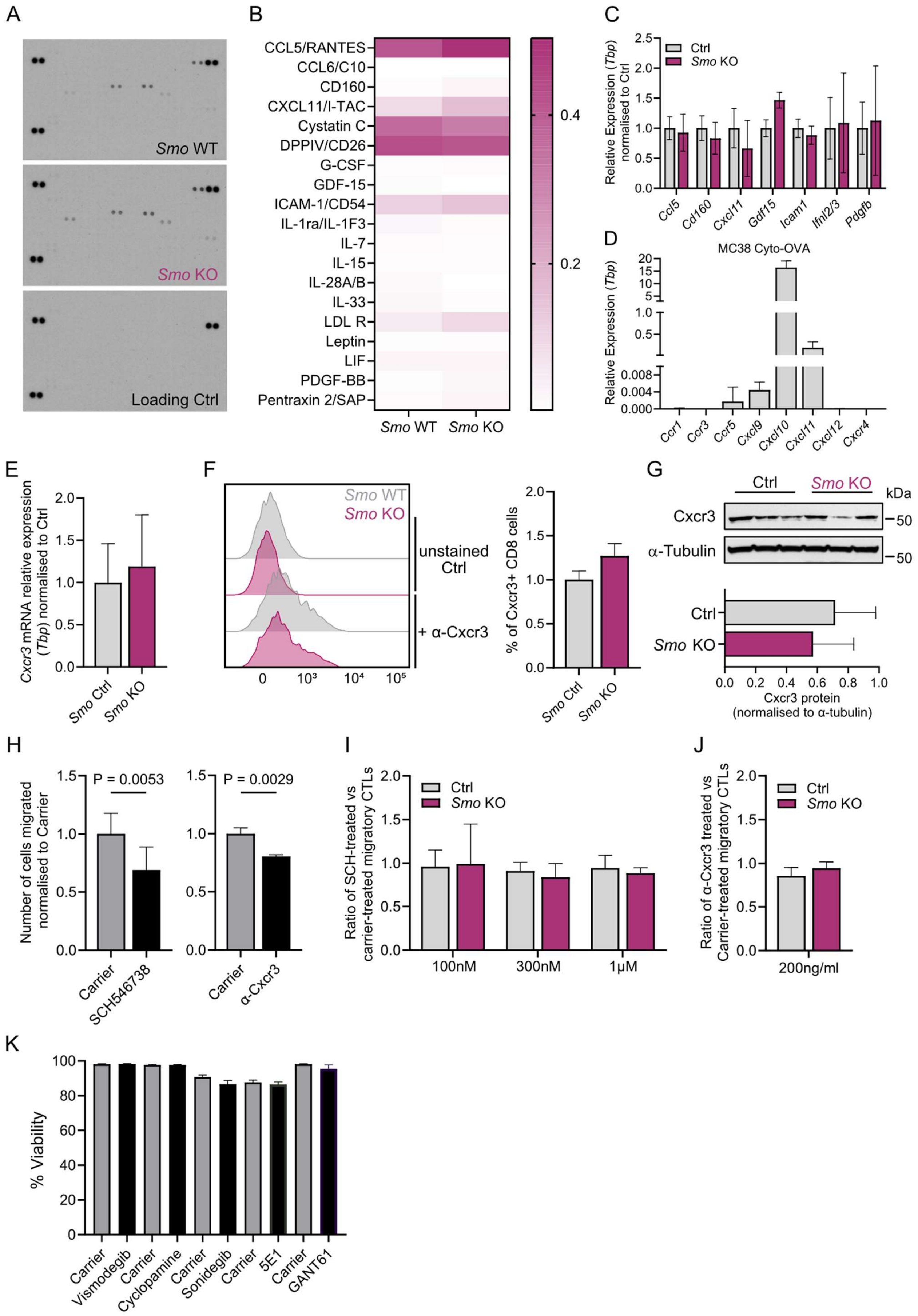
A
Smo WT
Smo KO
Loading Ctrl
B
CCL5/RANTES
CCL6/C10
CD160
CXCL11/I-TAC
Cystatin C
DPPIV/CD26
G-CSF
GDF-15
ICAM-1/CD54
IL-1ra/IL-1F3
IL-7
IL-15
IL-28A/B
IL-33
LDL R
Leptin
LIF
PDGF-BB
Pentraxin 2/SAP
Smo WT
Smo KO
0.4
0.2
C
Ctrl
Smo KO
Relative Expression (Tbp) normalised to Ctrl
Ccl5
Cd160
Cxcl11
Gdf15
Icam1
Ifnl2/3
Pdgfb
D
MC38 Cyto-OVA
Relative Expression (Tbp)
Ccr1
Ccr3
Ccr5
Cxcl9
Cxcl10
Cxcl11
Cxcl12
Cxcr4
E
Cxcr3 mRNA relative expression (Tbp) normalised to Ctrl
Smo Ctrl
Smo KO
F
Smo WT
Smo KO
unstained Ctrl
+ α-Cxcr3
% of Cxcr3+ CD8 cells
Smo Ctrl
Smo KO
G
Ctrl
Smo KO
kDa
Cxcr3
α-Tubulin
50
50
Ctrl
Smo KO
Cxcr3 protein (normalised to α-tubulin)
H
P = 0.0053
P = 0.0029
Number of cells migrated normalised to Carrier
Carrier
SCH546738
Carrier
α-Cxcr3
I
Ctrl
Smo KO
Ratio of SCH-treated vs carrier-treated migratory CTLs
100nM
300nM
1μM
J
Ctrl
Smo KO
Ratio of α-Cxcr3 treated vs Carrier-treated migratory CTLs
200ng/ml
K
% Viability
Carrier
Vismodegib
Carrier
Cyclopamine
Carrier
Sonidegib
Carrier
5E1
Carrier
GANT61

Figure S4: **Genetic loss of *Smo* in cytotoxic CD8 cells does not affect general cytokine production profiles or response to tumor chemoattractants via the Cxcr3-Cxcl9/10/11 axis.**

CTLs were generated from *GzmB-ERT2Cre/ROSAtdTom Smo*$^{fl/fl}$ (KO) and *Smo*$^{+/+}$ or *Smo*$^{f/+}$ (Ctrl) mice and used for downstream assays shown in (**A-C, E-G, I-K**) between d7/8 of *ex vivo* culture.

(A) Profiler XL array for cytokines expressed by *Smo*$^{fl/fl}$ (KO) or *Smo*$^{+/+}$ (Ctrl) CTLs on d8 lysed cell pellets.

(B) Quantification of signal intensity from (**A**). The mean of two technical repeats is shown in a single-gradient heatmap. One independent experiment, n=1 for *Smo* WT and n=1 for *Smo* KO.

(C) mRNA expression levels of highly expressed targets from the XL profiler array (**A, B**) assessed by qRT-PCR with *Tbp* as a housekeeping gene. n=4 for *Smo* Ctrl and n=3 for *Smo* KO, one independent experiment, mean ± SD.

(D) mRNA levels of key chemokines expressed by MC38-OVA cells. Four pellets were included and run in triplicates and *Tbp* was used as a housekeeping gene. One independent experiment, mean ± SD.

(E) mRNA expression levels of *Cxcr3* assessed by qRT-PCR, n=4 for *Smo* Ctrl and n=3 for *Smo* KO, one independent experiment, unpaired t-test showed no significant difference, P=0.7724, mean ± SD.

(F) Left: Representative flow cytometry histograms for Cxcr3 staining. Right: Percentage of Cxcr3+ cells out of CD3+CD8+ cells on d8. n=7 for *Smo* Ctrl and n=7 for *Smo* KO, two independent experiments, unpaired t-test showed no significant difference, P=0.1426, mean ± SD.

(G) Cxcr3 protein expression analysed by protein immunoblot with α-Tubulin used as a loading control. One independent experiment, n=3 for *Smo* Ctrl and n=3 for *Smo* KO. For the quantification panel (bottom), mean + SD is shown, P=0.5408.

(H) Transwell assays with Cxcl10 and Cxcl11-supplemented T cell media at the bottom well and d7/8 CTLs in drug-containing media in the insert wells. After 6hrs, cells from the bottom well were collected for flow cytometric analysis. SCH546738 (Cxcr3 antagonist)

100nM, n=7, two independent experiments. α-Cxcr3 200ng/ml, n=11, four independent experiments. For each drug paired t-tests were performed and mean ± SEM is shown.

(I) Transwell assays as in (**H**). Ratios of SCH546738-treated compared to carrier-treated CTLs is shown for each concentration. For 100nM, n=4 for *Smo* Ctrl and n=3 for *Smo* KO from two independent experiments. For 300nM and 1μM, n=4 for *Smo* Ctrl and n=4 for *Smo* KO, one independent experiment, mean ± SD.

(J) Transwell assays as in (**H**). Ratios of α-Cxcr3-treated (200ng/ml) compared to carrier-treated CTLs is shown. Two independent experiments, n=7 for *Smo* Ctrl and n=7 for *Smo* KO, mean ± SD.

(K) Viability of murine CD8+ cells after treatment with the drugs used for transwell assays in **Fig. 4B, C.**

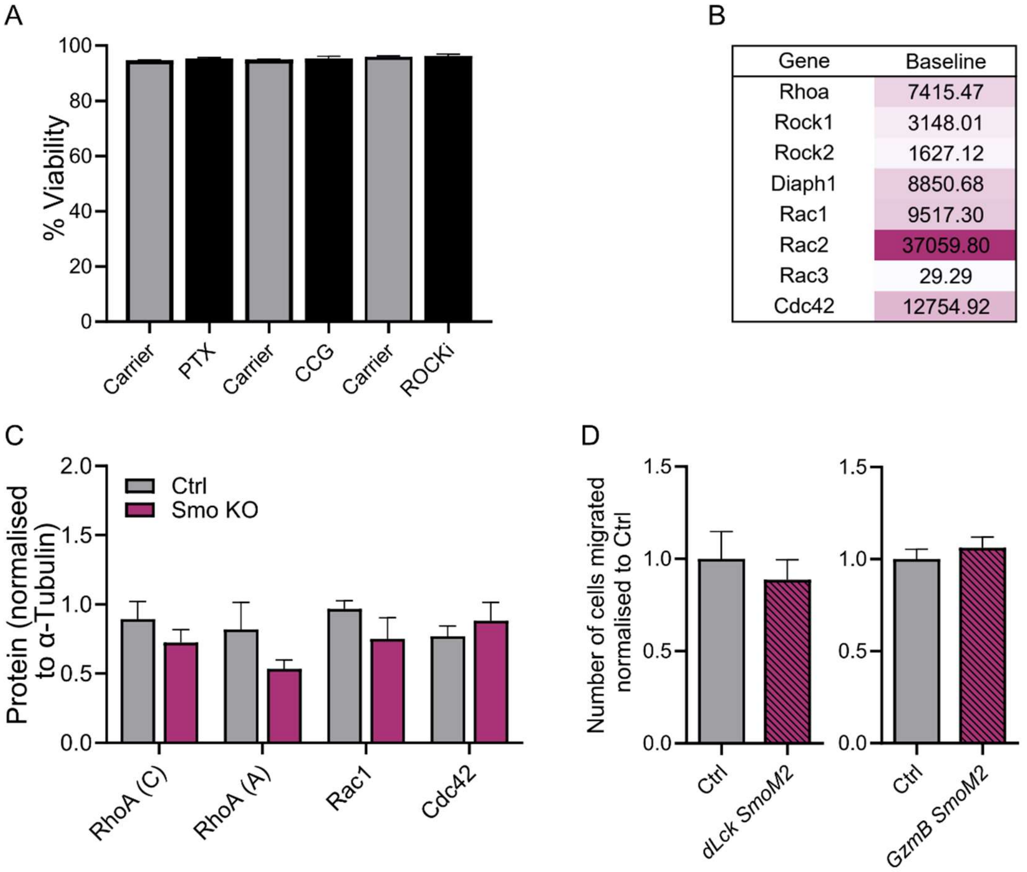

Figure S5: ***Smo* deletion does not affect steady state levels of RhoA, Rac1, and Cdc42 and *SmoM2* mutation has no effect in T cell migration *in vitro*.**

(A) Viability of CD8+ T cells after 8hr treatment with the drugs used for transwell assays in **Fig. 5B, C, F**. Two independent experiments, n=6 mice, mean + SEM is shown.

(B) mRNA expression of small GTPases determined by bulk RNASeq performed on murine CTLs on day 6 post stimulation, n=7 mice.

(C) Representative experiment of quantification of protein levels of SDS-PAGE gels shown in **Fig. 5D**. Values are normalised to loading control, α-Tubulin. Two separate antibodies were tested for RhoA, one form Cytoskeleton (C) and one from Abcam (A). Three independent experiments, n=6 for $Smo^{fl/+}$ (Ctrl), n=6 for $Smo^{fl/fl}$ (KO), mean + SD is shown.

(D) Transwell assays with cells from SmoM2 transgenic mice crossed to either *dLck* or *GzmB* promoters. SmoM2 is a dominant active mutation of Smo, originally identified in exon 9 of a pair of BCC patients and leads to a G-to-T transversion at base pair 1,604

(M2), changing codon 535 from Trp to Leu. Cxcl10 and Cxcl11-supplemented T cell media was dispensed at the bottom well and d7/8 CTLs in the insert wells. After 6hrs, cells from the bottom well were collected for flow cytometric analysis. *dLck-Cre SmoM2* n=5, Ctrl n=5, two independent experiments; *GzmB-ERT2Cre SmoM2* n=4, Ctrl n=4, two independent experiments, mean ± SEM is shown.

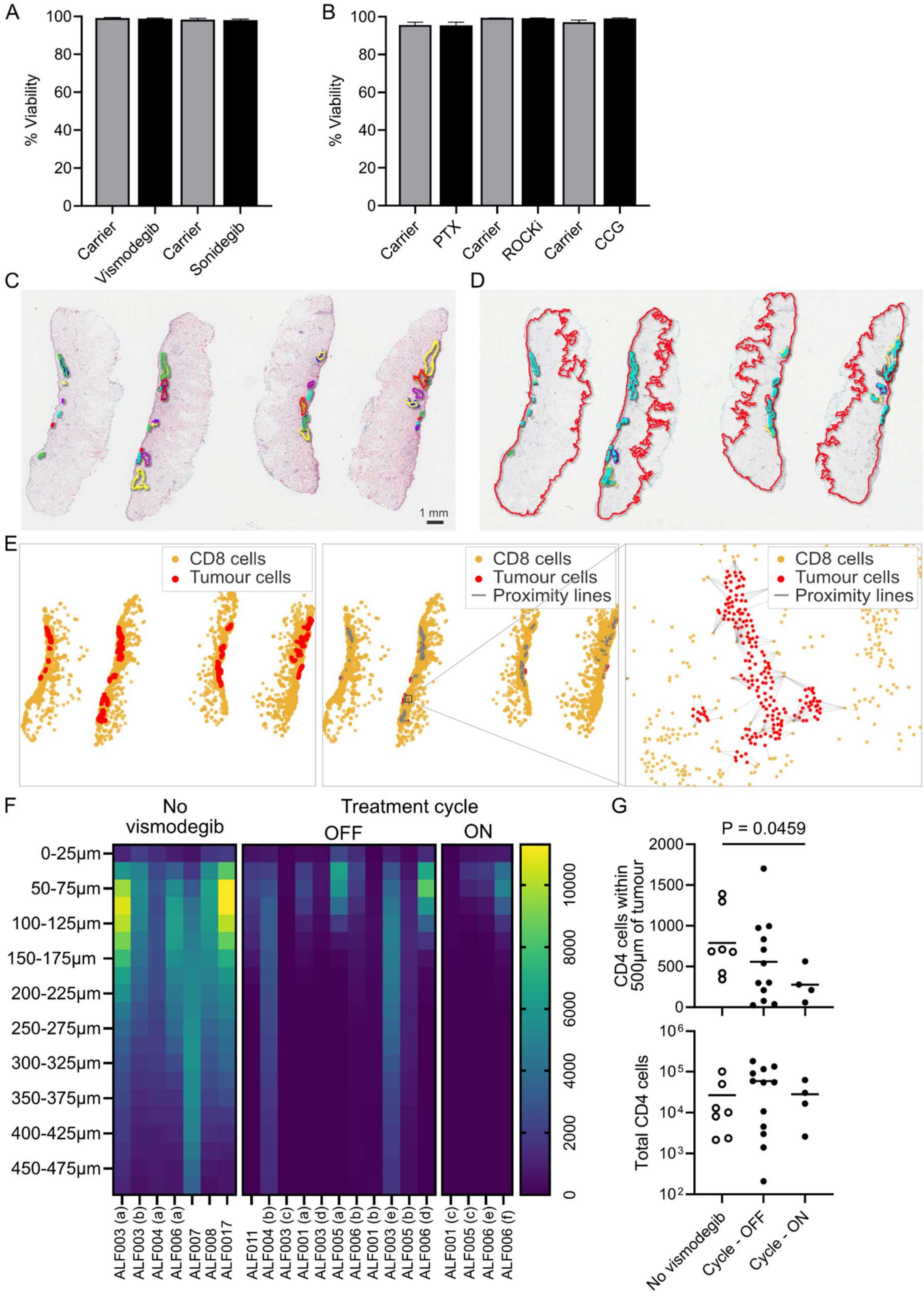
A
% Viability
100
80
60
40
20
0
Carrier
Vismodegib
Carrier
Sonidegib
B
% Viability
Carrier
PTX
Carrier
ROCKi
Carrier
CCG
C
1 mm
D
E
CD8 cells
Tumour cells
Proximity lines
F
No vismodegib
Treatment cycle
OFF
ON
0-25μm
50-75μm
100-125μm
150-175μm
200-225μm
250-275μm
300-325μm
350-375μm
400-425μm
450-475μm
10000
8000
6000
4000
2000
0
ALF003 (a)
ALF003 (b)
ALF004 (a)
ALF006 (a)
ALF007
ALF008
ALF0017
ALF011
ALF004 (b)
ALF003 (c)
ALF001 (a)
ALF003 (d)
ALF005 (a)
ALF006 (b)
ALF001 (b)
ALF003 (e)
ALF005 (b)
ALF006 (d)
ALF001 (c)
ALF005 (c)
ALF006 (e)
ALF006 (f)
G
P = 0.0459
CD4 cells within 500μm of tumour
2000
1500
1000
500
0
Total CD4 cells
10^6
10^5
10^4
10^3
10^2
No vismodegib
Cycle - OFF
Cycle - ON

Figure S6: Image analysis of BCC biopsies shows diminished CD4 T cell infiltration upon vismodegib treatment.

(A) Viability of human CD8+ T cells after treatment with the drugs used for transwell assays in **Fig. 6A**.

(B) Viability of human CD8+ T cells after treatment with the drugs used for transwell assays in **Fig. 6B**. Only the highest concentration (0.32mM) tried for CCG215022 is shown.

(C-E) Workflow of imaging analysis for human BCC sections.

(C) Example of annotation of individual tumor margins in consecutive H&E-stained BCC biopsies performed by a clinical histopathologist on which CD8 and CD4 proximity analysis was performed.

(D) Consecutive section of (**C**) stained with antibodies against human CD8a with classifiers for tumor regions (light blue) shown (red margins indicate the tissue area excluding adipose tissues).

(E) Mask of CD8 T cells in yellow and tumor cells in red with every dot representing one cell (left) throughout the whole tissue area. Same mask with proximity lines drawn in grey between each BCC cell and its nearest CD8 T cell neighbour within 500 μm outwards of the tumor margin (middle). Magnification of box region shown (right).

(F) Proximity analysis of each BCC cell to its closest neighbouring CD4 T cell (analysis workflow similar to one shown in **C-E**). Heatmap indicates the number of BCC tumor cells within the indicated distance brackets. n=7 BCCs before vismodegib treatment, n=11 BCCs during treatment cycle ("off") and n=4 BCCs during treatment cycle ("on"), originating from nine patients in total. BCCs during treatment cycle ("off") are ordered according to length of time since last vismodegib dose, from longest (*left*) to shortest (*right*). Detailed dates of sample collection, excision site and duration of treatment regime is shown in **Table S2**.

(G) Numbers of CD4T cells found within 500 μm of the tumor margins (top) and total numbers of CD4 T cells across the entire section (bottom) in the same excisions shown in **(F)**.

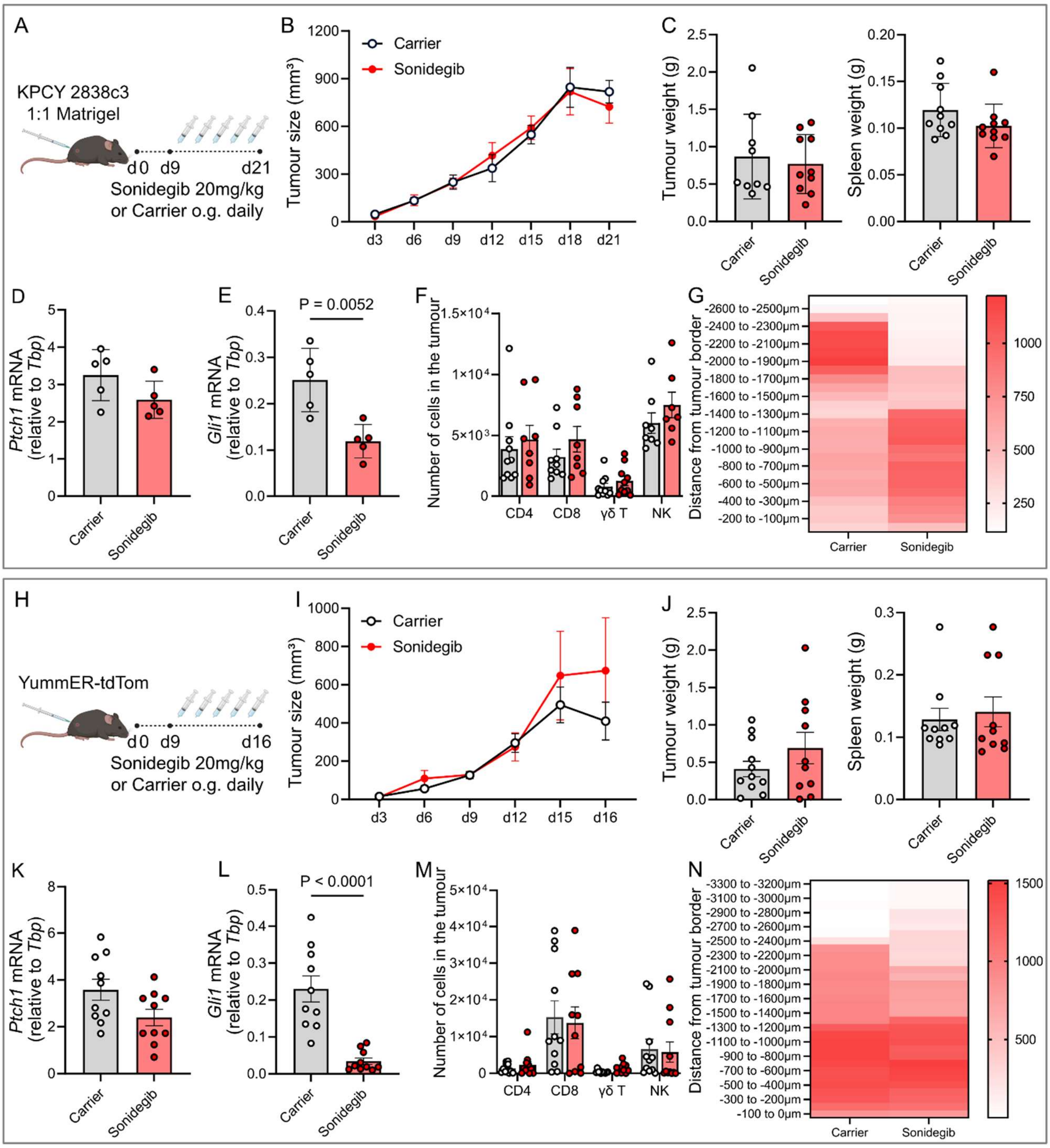

Figure S7: Sonidegib treatment fails to reduce the tumor burden in murine models of pancreatic cancer or melanoma.

(A) Experimental design. C57BL/6J wildtype animals were subcutaneously injected with 1×10^6 KPCY-2838c3 pancreatic cancer cells on d0 at a 1:1 ratio with matrigel. On d9,

mice were stratified into two equal groups according to tumor size. Between d9 and d21, mice were treated daily with 20mg/kg sonidegib or carrier control by oral gavage.

(B) Tumor dimensions as established by caliper measurements. Two independent experiments, n=10 for carrier-treated mice, n=10 for sonidegib-treated mice, ordinary two-way ANOVA with Geisser-Greenhouse correction, mean ± SEM.

(C) Tumor and spleen weight at endpoint (d21). Two independent experiments, n=10 for carrier-treated mice, n=10 for sonidegib treated mice, unpaired Mann-Whitney test, mean ± SEM.

(D, E) mRNA levels of *Ptch1* and *Gli1* in the small intestine. One independent experiment, n=5 for for carrier-treated mice, n=5 for sonidegib treated mice, unpaired Mann-Whitney test, mean ± SEM.

(F) Enumeration of immune subsets in the tumor by flow cytometry at endpoint (d21), normalised to tumor weight. Two independent experiments, n=10 for carrier-treated mice, n=7 for sonidegib treated mice, multiple unpaired t-tests, mean ± SEM.

(G) Mean of number of CD8+ cells/ mm^2 in 100µm-wide zones from the tumor surface (-100 to 0 µm) to the tumor centre (-2600 to -2500 µm) as quantified by HALO software (**Fig. S2A**). Two independent experiments, n=10 for carrier treatment mice, n=10 for sonidegib-treated mice. Mean is shown in a single gradient heatmap.

(H) Experimental design. C57BL/6J wildtype animals were subcutaneously injected with 1×10^6 YummER-tdTom melanoma cells on d0. On d9, mice were stratified into two equal groups according to tumor size. Between d9 and d16, mice were treated daily with 20mg/kg sonidegib or carrier control by oral gavage.

(I) Tumor dimensions as established by caliper measurements. Two independent experiments, n=12 for carrier-treated mice, n=13 for Sonidegib treated mice, ordinary two-way ANOVA with Geisser-Greenhouse correction, mean ± SEM.

(J) Tumor and spleen weight at endpoint (d16). Two independent experiments, n=12 for carrier-treated mice, n=10 for sonidegib treated mice, unpaired Mann-Whitney test, mean ± SEM.

(K, L) mRNA levels of *Ptch1* and *Gli1* in the small intestine. Two independent experiments, n=10 for carrier-treated mice, n=10 for sonidegib treated mice, unpaired Mann-Whitney test, mean ± SEM.

(M) Flow cytometry enumeration of immune subsets in the tumor at endpoint (d21), normalised to tumor weight. Two independent experiments, n=10 for carrier-treated mice, n=10 for sonidegib treated mice, unpaired Mann-Whitney test, mean ± SEM.

(N) Mean of number of CD8+ cells/ mm^2 in 100µm-wide zones from the tumor surface (-100 to 0 µm) to the tumor centre (-3300 to -3200 µm) as quantified by HALO software. Two independent experiments, n=9 for carrier treatment mice, n=9 for sonidegib-treated mice. Mean is shown in a single gradient heatmap.

Patient	Sample	Status	Date of sample collection	Last treatment window (patient off/on vismo) before excision	Last dosing date	Days since Vismodegib	Site	Tumour Type
**ALF003**	**a**	Gorlin	29/11/2015	not commenced vismodegib	-	-	Nose/forehead above eyebrow/jaw line	tumour
**ALF003**	**b**	Gorlin	17/10/2015	not commenced vismodegib	-	-	Right nasolabial fold	tumour
**ALF004**	**a**	Cancer	08/09/2017	not commenced vismodegib	-	-	Right uppper lip	tumour
**ALF006**	**a**	Cancer	03/12/2012	not commenced vismodegib	-	-	Scalp	tumour
**ALF007**		Cancer	22/11/2017	not commenced vismodegib	-	-	Left alar base/upper lip	tumour
**ALF008**		Cancer	27/09/2016	not commenced vismodegib	-	-	Left ear and neck	SCC
**ALF017**		Cancer	17/12/2020	not commenced vismodegib	-	-	Right thigh	tumour
**ALF011**		Gorlin	15/10/2020	10.1.17-01.02.2017	01/02/2017	1352	Right upper chest	BCC
**ALF004**	**b**	Cancer	17/05/2021	1.1.2017-1.4.2018	01/04/2018	1142	Superior right mastoid	tumour
**ALF003**	**c**	Gorlin	15/09/2021	22.04.2021 - 1.7.2021	01/07/2021	76	Vertex left scalp	sBCC
**ALF001**	**a**	Gorlin	04/05/2023	3.5.2023-8.3.2023	08/03/2023	57	Right scalp, zygoma, temple	tumour/ sBCC
**ALF003**	**d**	Gorlin	17/05/2023	1.2.2023 -31.3.2023	31/03/2023	47	Right neck & jaw	tumour
**ALF005**	**a**	Gorlin	14/10/2021	1.6.2021 - 1.9.2021	01/09/2021	43	Skin tissue T1	tumour
**ALF006**	**b**	Cancer	23/01/2017	14.9.2016-14.12.2016	14/12/2016	40	Dorsal right hand	SCC
**ALF001**	**b**	Gorlin	15/02/2017	19.10.2016 - 9.1.2017	09/01/2017	37	Left ear	sBCC
**ALF003**	**e**	Gorlin	15/12/2021	23.9.2021-24.11.2021	24/11/2021	21	Scalp- Mohs' Procedure	tumour
**ALF005**	**b**	Gorlin	15/02/2022	3.11.21-26.1.22	26/01/2022	20	Neck	tumour
**ALF006**	**c**	Cancer	11/03/2015	1.9.2014 - 19.02.2015	19/02/2015	20	Right forehead, ear and scalp	tumour
**ALF006**	**d**	Cancer	14/10/2015	9.4.2015 - 02.10.2015	25/09/2015	19	Scalp	tumour/sBCC
**ALF001**	**c**	Gorlin	28/09/2017	23.8.2017 - 12.11.2017	-	On	Right Eyebrow/supraorbital area	sBCC
**ALF005**	**c**	Gorlin	25/07/2023	1.6.2023 - 1.9.2023	-	On	Mohs left eye lid/right upper lip/right eyebrow	tumour
**ALF006**	**e**	Cancer	06/04/2023	22.03.2023 -17.5.23	-	On	Right posterior scalp	sBCC
**ALF006**	**f**	Cancer	06/04/2023	22.03.2023 -17.5.23	-	On	Right jawline	tumour

Table S2: Clinical information of BCC biopsies.

Details of BCC biopsies: date of excision, last treatment window before excision, last dosing date, time lapsed in days from last vismodegib dose, excision site and tumor type are shown. For each patient, separate BCCs excised over time are labelled a, b, c, … in chronological order. Gorlin status identifies patients with germline *PTCH1* mutations. Tumor types are defined as tumor (BCC), SCC (Squamous Cell Carcinoma) and sBCC (superficial BCC).

Smal molecule	Concentration	Carrier	Cat no.	Supplier
Vismodegib	5µM	DMSO	V-4050	LC Labs
Cyclopamine	5µM	ETOH	J61528	Thermo
Sonidegib	10µM	PEG/Dextrose/Water	S-4699	LC Labs
Anti-Hh ligand 5E1	10µg/ml	PBS	Ab01175-23-0	2Bscientific
GANT61	5µM	ETOH	1892-5	Cambridge Bioscience
SCH546738	100nM-1µM	DMSO	HY-10017	Cambridge Bioscience
Anti-Cxcr3	200 ng/ml	0.09% NaN_3 in PBS	126516	Biolegend
Pertussis Toxin	100ng/ml	Glycerol	P2980	Sigma
Rho kinase inhibitor	1µM	Water	555552	Calbiochem
CCG215022	0.032-0.32 mM	DMSO	S6621	Universal biologicals

Table S3: Small molecules used for migration assays.

Probe	Cat no.	Exon Boundary
CD3ε	Mm00599684_g1	6-7
Tbp	Mm00446973_m1	4-5
Smo	Mm01162705_m1	2-3
Ptch1	Mm00436026_m1	17-18
Gli1	Mm00494654_m1	11-12
Ccl5	Mm01302427_m1	1-2
Ccr1	Mm01216147_m1	1-2
Ccr3	Mm01216172_m1	1-2
Ccr5	Mm01216171_m1	1-2
Cd160	Mm00444461_m1	2-3
Cxcl9	Mm00434946_m1	2-3
Cxcl10	Mm00445235_m1	1-2
Cxcl11	Mm00444662_m1	1-2
Cxcl12	Mm00445553_m1	2-3
Cxcr4	Mm01292123_m1	1-2
Icam1	Mm00516023_m1	2-3
Ifn2/3	Mm04204158_gH	4-5
Gdf15	Mm00442228_m1	1-2
Pdgfb	Mm00440677_m1	4-5
PTCH1	Hs00181117_m1	19-20
GLI1	Hs01110766_m1	10-11
ACTINB	Hs01060665_g1	2-3

Table S4: Probes used for qRT-PCR.

Target	Clone	Reactivity	Type	Dilution	Cat no.	Supplier
RhoA	Monoclonal	Rabbit anti-mouse	Primary	1: 1,000	GL01C	Cytoskeleton
RhoA	Monoclonal	Rabbit anti-mouse	Primary	1: 5,000	ab187027	Abcam
Rac1	Monoclonal	Rabbit anti-mouse	Primary	1: 1,000	GL07	Cytoskeleton
Rac1	polyclonal	Rabbit anti-mouse	Primary	1: 20,000	ab155938	Abcam
Cxcr3	polyclonal	Rabbit anti-mouse	Primary	1: 500	ab71864	Abcam
Cdc42	4B8	Mouse anti-mouse	Primary	1: 250	ACD03	Cytoskeleton
α-Tubulin	DM1A	Chicken anti-mouse	Primary	1: 2,000	3873S	Cell Signalling
HRP conjugate	polyclonal	Goat anti-mouse	Secondary	1:15,000	P0447	Agilent Dako
HRP conjugate	polyclonal	Goat anti-rabbit	Secondary	1: 10,000	P0448	Agilent Dako

Table S5: Antibodies used for protein immunoblot.

Target	Clone	Reactivity	Fluorochrome	Dilution	Cat no.	Supplier
CD3ε	145-2C11	Mouse	BUV395	1:50	563565	BD
CD3ε	145-2C11	Mouse	FITC	1:50	100306	Biolegend
CD8a	53-6.7	Mouse	BUV737	1:200	563786	BD
CD4	53-6.7	Mouse	BV605	1:200	100548	Biolegend
CD44	IM7	Mouse/Human	BV605	1:400	103047	Biolegend
CD44	IM7	Mouse/Human	BV650	1:400	103049	Biolegend
CD44	IM7	Mouse/Human	BV785	1:400	103059	Biolegend
CD45	30-F11	Mouse	AF700	1:200	103128	Biolegend
CD45	30-F11	Mouse	PE-Cy7	1:200	103114	Biolegend
CD62L	MEL-14	Mouse	BV510	1:200	104441	Biolegend
CD27	LG.7F9	Mouse/Rat/ Human	PE-Cy7	1:100	25-0271-82	eBioscience
CD49b	HMα2	Mouse	BV650	1:100	740496	BD
Sca1	D7	Mouse	BV785	1:200	108139	Biolegend
CD150	TC15-12F12.2	Mouse	BV711	1:50	115941	Biolegend
TCRγδ	GL3	Mouse	APC	1:200	118116	Biolegend
TIM3	RMT3-23	Mouse	BV605	1:100	119721	Biolegend
TCRβ	H57-597	Mouse	BV711	1:100	109243	Biolegend
NK1.1	PK136	Mouse	BUV395	1:100	564144	BD
NK1.1	PK136	Mouse	AF700	1:100	108730	Biolegend
CD11b	M1/70	Mouse/Human	BV605	1:100	101237	Biolegend
CXCR3	CXCR3-173	Mouse	PE-Cy7	1:50	126516	Biolegend
IL7Rα	A7R34	Mouse	BV711	1:50	135024	Biolegend
KLRG	2F1/KLRG 1	Mouse/Human	BV711	1:100	138427	Biolegend
LAG3	eBioC9B7 W (C9B7W)	Mouse	APC	1:100	17-2231-82	eBioscience
CD69	H1.2F3	Mouse	PE	1:200	104507	Biolegend
PD-1	RMP1-30	Mouse	BV421	1:50	109121	Biolegend
GzmB	GB11	Mouse/Human	AF647	1:100	515406	Biolegend
Perforin	S16009A	Mouse	FITC	1:100	154310	Biolegend
TNFα	MP6-XT22	Mouse	PE	1:100	506306	Biolegend

IFNγ	XMG1.2	Mouse	PE	1:100	505808	Biolegend
CD45RA	HI100	Human	APC	1:400	304112	Biolegend
CCR7	G043H7	Human	PE-Cy7	1:400	353208	Biolegend
CD8a	HIT8a	Human	FITC	1:400	300906	Biolegend
CD3	UCHT1	Human	PE	1:400	300408	Biolegend

Table S6: Antibodies used for flow cytometry.

Target	Catalogue No.	Dilution/Conc.	Retrieval	Modifications
CD3	Dako, A0452	1:1000	Tris EDTA, 20’	No Post Primary, DAB Enhancer, 2’ Haematoxylin
CD8	Cell Signaling Technology,98941	1:200	Tris EDTA, 20’	Protein Block, No Post Primary, DAB Enhancer
B220	R&D Systems, MAB1217	0.6666 ug/ml	Sodium Citrate, 10’	Protein Block, Anti-Rat Secondary, DAB Enhancer
CD11c	Cell Signaling Technology, 97585	0.0625 ug/ml	Tris EDTA, 20’	Protein Block, No Post Primary, DAB Enhancer
F4/80	Serotec, MCA497	1:20	Sodium Citrate, 20’	Protein Block, Anti-Rat Secondary, DAB Enhancer
ASMA	Abcam, ab5694	1:500	Tris EDTA, 10’	No Post Primary, DAB Enhancer
CD31	Cell Signaling, 77699	1:100	Tris EDTA, 20’	Protein Block, No Post Primary, DAB Enhancer
Ly6G	BioLegend, 127601	0.6667 ug/ml	Sodium Citrate, 20’	Protein Block, Anti-Rat Secondary, DAB Enhancer
PD1	Sino Biological Inc., 50124-RP01	1:400	Sodium Citrate, 20’	No Post Primary, DAB Enhancer
CD4	Dako, M7310	1:50	Tris EDTA, 20’	DAB Enhancer
CD8	Neomarkers, RM-9116-S	1:100	Tris EDTA, 20’	Protein Block, DAB Enhancer
CD20	Novocastra, NCL-L-CD20-L26 (CD20-L26-L-CE)	0.95ug/ml	Tris EDTA, 20’	Protein Block, DAB Enhancer
CD44	Atlas, HPA005785	1:800	Sodium Citrate, 30’	300mM Tris Diluent, No Post Primary, DAB Enhancer
CD45	Dako, M0701	1.5 ug/ml	Tris EDTA, 20’	Protein Block, DAB Enhancer
CD68	Novocastra, NCL-L-CD68 (CD68-L-CE)	1:50	Tris EDTA, 20’	DAB Enhancer
PD-L1	Cell Signaling Technologies, 13684	8.74 ug/ml	Tris EDTA, 20’	Protein Block, No Post Primary, DAB Enhancer
pan-CK(AE1/3)	Dako, M3515	1:200	Sodium Citrate, 20’	DAB Enhancer

Table S7: Antibodies used for immunohistochemistry.
